# Global, regional, and national burdens of intraocular foreign bodies in children and adolescents from 1990 to 2019: a trend analysis

**DOI:** 10.1186/s12889-023-17401-0

**Published:** 2023-12-12

**Authors:** Hang Ren, Bo Jiang, Gaoqin Liu, Peirong Lu

**Affiliations:** Department of Ophthalmology, the First Affiliated Hospital of Soochow University, 188 Shizi Street, 215006 Suzhou, Jiangsu China

**Keywords:** Intraocular foreign bodies, Global burden of Disease Study, Children, Adolescents, Trend

## Abstract

**Background:**

This study aimed to evaluate trends in global, regional, and national burdens of intraocular foreign bodies among children and adolescents (aged 0 − 19 years) between 1990 and 2019 according to age, sex, and socio-demographic index.

**Methods:**

This study obtained data from the Global Burden of Disease Study 2019 and evaluated the number of cases, rates per 100,000 persons, and average annual percentage changes among children and adolescents. The annual percentage changes in the incidence and years lived with disability rates across various age groups were investigated using joinpoint software.

**Results:**

For intraocular foreign bodies in children and adolescents, the incidence and year lived with disability rates decreased in all age groups between 1990 and 2019. However, the number of incident cases and years lived with disability increased from 1091.94 [95% uncertainty interval (UI), 610.91–1839.52] and 89,245 (95% UI, 6.65–18.67) in 1990 to 1134.85 (95% UI, 665.01–1867.50) and 92,108 (95% UI, 32,052–192,153) in 2019, respectively. Age was positively correlated with the number of cases, incidence, and years lived with disability rates. However, there were significant decreases in both the incidence and years lived with disability rates among children and adolescents, especially in the 15–18 years age group, males, and most high-income regions. Notably, the incidence and years lived with disability rates were significantly decreased in middle and high-middle socio-demographic index regions but were increased in low and low-middle socio-demographic index regions.

**Conclusions:**

Despite the remarkable progress between 1990 and 2019 in reducing the global burden of intraocular foreign bodies, there has been an increase in the number of cases, with substantial disparity across age groups, sexes, regions, and countries. Our results could inform more effective strategies for reducing the burden among children and adolescents.

**Supplementary Information:**

The online version contains supplementary material available at 10.1186/s12889-023-17401-0.

## Background

Open-globe injury (OGI) is a leading cause of unilateral visual impairment caused by blunt trauma or sharp object injury and is characterised by full thickness break of the cornea or sclera with or without exposure of eye content to the environment. Its subtypes include penetration, perforation, rupture, and intraocular foreign bodies (IOFBs) [1]. IOFBs are a major public health problem, accounting for up to 41% of all OGIs [2]. They affect not only the individual but also the socioeconomic fabric of the community. Affected children and adolescents not only suffer from prolonged visual impairment but also decreased educational opportunities. One-third of the global economic cost of preventing and treating visual impairment and blindness is spent on children; however, nearly 50% of all causes are preventable or treatable, indicating that children need more focused health and medical services [3] .

Previous studies have reported the prevalence and incidence rate of IOFBs in the general population, without considering potential age-specific differences. A trend analysis of data from the Global Burden of Disease (GBD) Study 2017 showed that the global disability-adjusted life-years (DALYs) due to IOFBs increased by 43.7% between 1990 (n = 139,000) and 2017 (n = 202,000), and IOFBs mostly occur in patients aged 21–41 years [4, 5]. Further, 2019 GBD data revealed that the global incidence of IOFB increased from 35.79 million in 1990 to 46.63 million in 2019, representing an increase by 30.29%, while the age-standardised incidence rate decreased from 665.92 per 100,000 population in 1990 to 593.26 per 100,000 population in 2019, with an estimated annual percentage change of -0.93% [6]. Although there has been extensive research on the global epidemiology of IOFBs, there remain no age-specific data regarding IOFBs in children and adolescents aged 0 − 19 years.

Therefore, to gain insights into the global, regional, and national trends of IOFBs among children and adolescents—the age category for whom IOFBs bear the severest consequences—we aimed to use GBD 2019 data to evaluate case numbers, incidence rates and years lived with disability (YLD) rates according to age, sex, and socio-demographic index (SDI). The average annual percentage changes (AAPCs) in the incidence and YLD rates were estimated for different age groups between 1990 and 2019 using Joinpoint regression analysis. Our findings not only corroborate previous findings but also can guide future strategies for mitigating the socioeconomic and healthcare burden of IOFBs in children and adolescents.

## Methods

### Overview

GBD 2019 from the Global Health Data Exchange—which includes data regarding the global burden of 369 diseases and injuries, including IOFBs, in 21 GBD regions and 204 countries and territories from 1990 to 2019—was used to obtain repeated cross-sectional data [7]. The GBD 2019 global, age-specific, sex-specific, and region- and country-level incidence and YLD cases, as well as the corresponding incidence and YLD rates, of IOFBs from 1990 to 2019 were obtained for analysis. Since these data were freely available in public databases, ethical approval and informed consent were not required.

### Data extraction and variables

The Disease Modelling-Meta Regression version 2.1 was used to model the epidemiological outcomes of IOFBs in children and adolescents, which is a Bayesian meta-regression framework widely employed in GBD epidemiological modelling [8, 9]. Data regarding all children and adolescents with IOFBs in GBD 2019 were included. Specifically, data regarding IOFB indicators from both sexes in the four age groups as well as according to SDI, geographical location, and income level—as defined in GBD 2019—were collected. The results are presented as the number of cases, incidence rates, YLD rates, and AAPCs in incidence and YLD rates according to age, sex, region, and nation between 1990 and 2019 using 95% uncertainty intervals (UI) and 95% confidence intervals (CI). The 95% UI were determined by the 2.5th and 97.5th percentiles of 1,000 ordered draws according to the GBD algorithm [10]. Adolescents, as a life stage between childhood and adulthood, are affected by factors related to biological growth, social changes, and behavioural changes. According to the World Health Organization, adolescents are defined as those aged 10–19 years [11]. Similarly, we defined adolescents and children as those aged 10–19 years and 0–10 years, respectively. Further, we defined age subgroups as follows: preschool children (aged 0–4 years), older children (aged 5–9 years), young adolescents (aged 10–14 years), and older adolescents (15–19 years). Subsequently, children and adolescents were divided into various heterogeneous groups to obtain more detailed information regarding those life stages. The SDI represents the geometric average of 0–1 indices of the total fertility rate among women aged < 25 years, mean years of education in individuals aged > 14 years, and income per capita, where 1 represents the longest years of education, highest per capita income, and lowest fertility rate [7]. This study included five SDI quintiles: low, low-middle, middle, high-middle, and high. In total, based on geographical proximity and epidemiological similarity, 204 countries and regions were divided into 21 GBD regions.

### Statistical analysis

The incidence rates, YLD rates, and their AAPCs were calculated through linear regression analysis, with year and logarithm-transformed rates as the independent and dependent variables, respectively. The AAPC value, which represents the annual percentage change (increase, decrease, or no change), is used to summarise and compare trends that may not be constant within a given period [12]. For example, an AAPC of 0.1 indicates an annual increase rate of 0.1%. The AAPC values and their 95% CIs reflect the trends of rates [8]. We employed joinpoint regression analysis using the joinpoint statistical software version 4.9.0.0 (National Cancer Institute, Information Management Services, Inc., Calverton, MD, USA) to estimate annual percent changes (APCs) in the incidence and YLD rates according to age. Joinpoint software [13] is used to identify temporal trends in data and to connect several line segments by fitting the simplest model to the data on a logarithmic scale. These segments begin with 0 joinpoints (representing a straight line), followed by assessment of the statistical significance of the changes after adding more joinpoints along with their 95% CIs. With addition of more joinpoints, each jointpoint was tested using a Monte Carlo permutation method. The APC was computed as APCi = [(exp(β i)-1)] × 100, where β i represents the slope of the trend segment [14, 15]. All statistical analyses and data visualisations were performed using R version 4.2.2 (R Foundation for Statistical Computing, Vienna, Austria) and GraphPad Prism 9.0 (GraphPad Software, Inc., La Jolla, CA, USA). Statistical significance was set at *P* < 0.05.

## Results

### Global IOFB burden among children and adolescents

Globally, the number of incident cases and YLDs increased from 10.92 million (95% UI, 6.11–18.40) and 89,245 (95% UI, 6.65–18.67) in 1990 to 11.35 million (95% UI, 6.65–18.67) and 92,108 (95% UI, 32,052–192,153) in 2019, respectively. However, the incidence rates of IOFBs decreased from 480.28 (95% UI, 269.7–809.09) per 100,000 population in 1990 to 439.99 (95% UI, 257.83 to 724.04) per 100,000 population in 2019, with an AAPC of -0.3 (95% CI, -0.36 to -0.24). Moreover, the YLD rates declined from 3.93 (95% UI, 1.37–8.23) per 100,000 population in 1990 to 3.57 (95% UI, 1.24–7.45) per 100,000 population in 2019, with an AAPC of -0.32 (95% CI, -0.40 to -0.25) (Table 1).

**Table 1:** Incidence and Years Lived with Disability of Intraocular Foreign Bodies and Their Average Annual Percentage Changes from 1990 to 2019 at the Global and Regional Levels

	Incidence						YLDs					
	Number, 1990	Incidence rate (per 100 000 Population), 1990	Number, 2019	Incidence rate (per 100 000 Population), 2019	AAPC, 1990–2019	*P* Value	Number, 1990	YLD rate (per 100 000 Population), 1990	Number, 2019	YLD rate (per 100 000 Population), 2019	AAPC, 1990–2019	*P* Value
**Worldwide**	10,919,433 (6,109,109–18,395,169)	480.28 (268.7-809.09)	11,348,514 (6,650,092–18,674,962)	439.99 (257.83-724.04)	-0.3 (-0.36 - -0.24)	< 0.001	89,245 (31,164–187,100)	3.93 (1.37–8.23)	92,108 (32,052–192,153)	3.57 (1.24–7.45)	-0.32 (-0.4 - -0.25)	< 0.001
**Sex**												
Female	3,156,521 (1,836,702–5,359,272)	284.68 (165.65-483.34)	3,566,286 (2,142,281–5,829,099)	285.35 (171.41–466.4)	0 (-0.06–0.05)	0.007	26,182 (9031–54,795)	2.36 (0.81–4.94)	29,430 (10,240–61,343)	2.35 (0.82–4.91)	-0.02 (-0.07–0.04)	0.524
Male	7,762,912 (4,245,886–13,182,670)	666.48 (364.53-1131.79)	7,782,228 (4,497,899–12,798,809)	585.37 (338.32–962.7)	-0.44 (-0.53 - -0.36)	< 0.001	63,063 (21,690–133,302)	5.41 (1.86–11.44)	62,678 (21,884–130,745)	4.71 (1.65–9.83)	-0.47 (-0.57 - -0.36)	< 0.001
**Age Group**												
< 5 years	1,998,181 (1,100,613–3,310,588)	316.11 (174.12-523.73)	1,923,394 (1,084,866–3,065,851)	290.17 (163.67-462.53)	-0.31 (-0.37 - -0.26)	< 0.001	15,210 (4486–33,444)	2.41 (0.71–5.29)	14,618 (4412–32,398)	2.21 (0.67–4.89)	-0.32 (-0.37 - -0.26)	< 0.001
5–9 years	2,449,795 (1,252,697–4,845,036)	418.65 (214.08-827.98)	2,504,384 (1,274,876–4,792,559)	382.52 (194.73-732.02)	-0.32 (-0.37 - -0.27)	< 0.001	19,382 (5809–44,362)	3.31 (0.99–7.58)	19,728 (5815–44,774)	3.01 (0.89–6.84)	-0.34 (-0.39 - -0.29)	< 0.001
10–14 years	2,848,320 (1,165,020–5,597,355)	530.72 (217.08-1042.94)	3,104,852 (1,358,334–5,957,451)	483.48 (211.52-927.68)	-0.32 (-0.37 - -0.27)	< 0.001	23,479 (7278–54,898)	4.37 (1.36–10.23)	25,392 (8045–58,947)	3.95 (1.25–9.18)	-0.35 (-0.4 - -0.29)	< 0.001
15–19 years	3,623,135 (1,371,769–7,059,748)	697.29 (264-1358.68)	3,815,882 (1,651,123–7,138,158)	615.92 (266.51-1152.17)	-0.43 (-0.51 - -0.36)	< 0.001	31,174 (9700–73,641)	6 (1.87–14.17)	32,371 (10,484–74,411)	5.22 (1.69–12.01)	-0.48 (-0.56 - -0.41)	< 0.001
**SDI Region**												
High SDI	982,958 (581,844–1,636,881)	419.77 (248.48-699.03)	920,044 (539,886–1,550,763)	416.63 (244.48-702.25)	-0.05 (-0.14–0.04)	0.095	8112 (2849–16,840)	3.46 (1.22–7.19)	7610 (2697–15,760)	3.45 (1.22–7.14)	-0.04 (-0.13–0.05)	0.392
High-middle SDI	2,022,871 (1,050,765–3,554,529)	499.17 (259.29-877.12)	1,363,114 (758,977–2,308,801)	415.63 (231.42-703.98)	-0.63 (-0.74 - -0.52)	< 0.001	16,574 (5741–35,835)	4.09 (1.42–8.84)	11,037 (3787–23,050)	3.37 (1.15–7.03)	-0.66 (-0.79 - -0.53)	< 0.001
Middle SDI	3,998,935 (2,011,835–7,199,130)	521.42 (262.32–938.7)	3,280,570 (1,814,757–5,445,273)	445.4 (246.39-739.29)	-0.53 (-0.68 - -0.38)	< 0.001	33,124 (11,289–72,612)	4.32 (1.47–9.47)	26,796 (9245–55,935)	3.64 (1.26–7.59)	-0.57 (-0.76 - -0.39)	< 0.001
Low-middle SDI	2,702,729 (1,639,827–4,366,691)	473.67 (287.39-765.29)	3,325,798 (1,997,215–5,321,112)	478.28 (287.22-765.23)	0.03 (0–0.06)	< 0.001	21,642 (7313–44,466)	3.79 (1.28–7.79)	26,680 (9021–54,909)	3.84 (1.3–7.9)	0.03 (0–0.06)	0.052
Low SDI	1,207,661 (759,535–1,908,314)	408.86 (257.15-646.07)	2,453,484 (1,510,715–3,927,368)	410.99 (253.07-657.89)	0.02 (0.01–0.02)	< 0.001	9758 (3359–20,122)	3.3 (1.14–6.81)	19,940 (6860–40,913)	3.34 (1.15–6.85)	0.04 (0.03–0.04)	< 0.001
**South–East Asia, East Asia, and Oceania**												
East Asia	3,021,488 (1,045,646–6,160,962)	649.13 (224.65-1323.61)	1,264,477 (437,214–2,631,782)	406.73 (140.63-846.53)	-1.58 (-2.06 - -1.1)	< 0.001	25,622 (8356–60,668)	5.5 (1.8-13.03)	10,638 (3396–25,202)	3.42 (1.09–8.11)	-1.6 (-2.13 - -1.07)	< 0.001
Southeast Asia	540,439 (331,979–896,210)	244.23 (150.02–405)	578,368 (353,340–943,615)	256.55 (156.73-418.56)	0.17 (0.17–0.17)	< 0.001	4429 (1542–9093)	2 (0.7–4.11)	4739 (1640–9861)	2.1 (0.73–4.37)	0.17 (0.17–0.17)	< 0.001
Oceania	7141 (4560–11,381)	215.72 (137.75-343.83)	13,203 (8444–20,993)	216.14 (138.24-343.67)	0.01 (0–0.01)	< 0.001	57 (19–119)	1.71 (0.57–3.6)	104 (35–219)	1.71 (0.57–3.59)	0 (-0.01–0.01)	0.979
**Sub-Saharan Africa**												
Eastern Sub-Saharan Africa	397,225 (248,525–630,809)	359.05 (224.64-570.19)	820,397 (505,725–1,320,278)	367.03 (226.25-590.67)	0.08 (0.07–0.08)	0.008	3252 (1145–6684)	2.94 (1.04–6.04)	6742 (2365–13,774)	3.02 (1.06–6.16)	0.09 (0.08–0.1)	< 0.001
Central Sub-Saharan Africa	96,949 (61,828–154,603)	307.61 (196.17-490.53)	224,436 (140,820–362,928)	315.39 (197.89-510.01)	0.09 (0.08–0.09)	< 0.001	763 (255–1601)	2.42 (0.81–5.08)	1768 (586–3725)	2.48 (0.82–5.23)	0.09 (0.09–0.1)	< 0.001
Southern Sub-Saharan Africa	105,577 (65,429–173,852)	402.84 (249.65-663.36)	123,868 (76,518–204,007)	404.62 (249.95-666.39)	0.01 (0.01–0.02)	0.987	903 (328–1818)	3.45 (1.25–6.94)	1060 (388–2126)	3.46 (1.27–6.94)	0.01 (0–0.02)	0.003
Western Sub-Saharan Africa	388,268 (243,654–615,506)	361.71 (226.99-573.41)	931,431 (572,675–1,493,658)	375.16 (230.66-601.62)	0.13 (0.12–0.13)	< 0.001	3300 (1211–6656)	3.07 (1.13–6.2)	7940 (2929–15,986)	3.2 (1.18–6.44)	0.14 (0.13–0.14)	< 0.001
**South Asia**												
South Asia	2,962,069 (1,826,814–4,621,237)	540.46 (333.32–843.2)	3,969,242 (2,378,945–6,254,176)	571.5 (342.53–900.5)	0.19 (0.19–0.2)	< 0.001	23,331 (7660–48,462)	4.26 (1.4–8.84)	31,347 (10,299–65,105)	4.51 (1.48–9.37)	0.2 (0.2–0.21)	< 0.001
**Latin America and Caribbean**												
Caribbean	61,600 (37,746–97,710)	408.26 (250.17-647.59)	64,162 (38,917–102,729)	413.1 (250.56–661.4)	0.04 (0.03–0.05)	< 0.001	490 (166–1015)	3.25 (1.1–6.73)	508 (172–1059)	3.27 (1.1–6.82)	0.03 (0.02–0.04)	< 0.001
Central Latin America	403,051 (247,274–630,533)	489.78 (300.48-766.21)	435,175 (262,301–686,975)	497.64 (299.95-785.58)	0.05 (0.05–0.06)	< 0.001	3342 (1179–6755)	4.06 (1.43–8.21)	3599 (1267–7346)	4.12 (1.45–8.4)	0.04 (0.04–0.05)	< 0.001
Tropical Latin America	394,656 (238,422–622,468)	566.76 (342.4-893.92)	385,634 (231,270–606,133)	577.17 (346.14-907.19)	0.06 (0.06–0.07)	0.002	3372 (1235–6772)	4.84 (1.77–9.73)	3295 (1221–6690)	4.93 (1.83–10.01)	0.06 (0.06–0.07)	< 0.001
Andean Latin America	77,072 (48,256–122,569)	402.76 (252.18-640.52)	97,435 (59,890–154,868)	411.46 (252.91-653.99)	0.07 (0.07–0.08)	< 0.001	608 (201–1270)	3.18 (1.05–6.63)	766 (256–1596)	3.24 (1.08–6.74)	0.06 (0.06–0.07)	< 0.001
**North Africa and Middle East**												
North Africa and Middle East	774,613 (484,895–1,222,924)	429.64 (268.95-678.29)	983,885 (603,300–1,566,352)	430.03 (263.69-684.61)	0 (0–0.01)	0.018	6121 (2029–12,789)	3.39 (1.13–7.09)	7792 (2611–16,098)	3.41 (1.14–7.04)	0.01 (0–0.02)	0.02
**Central Europe, Eastern Europe, and Central Asia**												
Central Asia	124,107 (76,951–198,693)	393.47 (243.97-629.93)	135,463 (84,120–218,576)	398.62 (247.54–643.2)	0.04 (0.01–0.07)	0.189	973 (321–2041)	3.09 (1.02–6.47)	1061 (351–2225)	3.12 (1.03–6.55)	0.04 (0.01–0.07)	0.018
Eastern Europe	346,707 (211,996–568,170)	515.05 (314.93-844.04)	243,732 (149,777–403,525)	513.94 (315.82-850.89)	0 (-0.05–0.04)	0.019	2719 (903–5730)	4.04 (1.34–8.51)	1905 (631–4036)	4.02 (1.33–8.51)	-0.02 (-0.06–0.03)	0.498
Central Europe	159,933 (96,650–259,957)	414.76 (250.65-674.16)	96,939 (58,919–156,899)	414.35 (251.84-670.64)	-0.01 (-0.02–0.01)	0.014	1259 (415–2633)	3.26 (1.08–6.83)	761 (250–1591)	3.25 (1.07–6.8)	-0.02 (-0.03–0)	0.018
**High–income regions**												
Southern Latin America	53,384 (32,811–86,613)	275.56 (169.36-447.07)	55,740 (33,988–90,842)	279.13 (170.2-454.91)	0.04 (0.02–0.06)	0.032	422 (139–887)	2.18 (0.72–4.58)	440 (145–929)	2.2 (0.73–4.65)	0.04 (0.02–0.06)	< 0.001
Western Europe	333,509 (201,853–537,555)	338.92 (205.13-546.27)	310,575 (188,163–502,847)	336.66 (203.97-545.08)	-0.02 (-0.03 - -0.01)	0.619	2650 (883–5542)	2.69 (0.9–5.63)	2465 (818–5177)	2.67 (0.89–5.61)	-0.03 (-0.03 - -0.02)	< 0.001
North America	394,951 (209,858–689,146)	484.89 (257.65-846.08)	426,445 (230,031–748,622)	474.14 (255.76-832.35)	-0.11 (-0.41–0.19)	0.045	3359 (1207–7068)	4.12 (1.48–8.68)	3644 (1322–7711)	4.05 (1.47–8.57)	-0.1 (-0.37–0.18)	0.501
Australasia	25,610 (15,909–41,117)	408.14 (253.54-655.26)	28,985 (18,023–46,908)	402.24 (250.12-650.96)	-0.05 (-0.06 - -0.04)	0.029	202 (67–420)	3.22 (1.06–6.7)	228 (75–475)	3.17 (1.05–6.59)	-0.06 (-0.07 - -0.05)	< 0.001
Asia Pacific	251,084 (154,555–404,045)	498.34 (306.75-801.93)	158,923 (98,320–254,118)	492.07 (304.43-786.82)	-0.04 (-0.05 - -0.03)	0.022	2071 (727–4319)	4.11 (1.44–8.57)	1307 (459–2699)	4.05 (1.42–8.36)	-0.05 (-0.06 - -0.04)	< 0.001

AAPC = average annual percent change; YLDs = years lived with disability; SDI = sociodemographic index

### Age-specific IOFB burden among children and adolescents

The trends in the incidence and YLDs for IOFBs across all ages (among children and adolescents) from 1990 to 2019 are presented in Table 1. Globally, the number of incident cases and YLDs in the < 5-year age group decreased from 1990 to 2019, whereas it increased in the other three groups. Between 1990 and 2019, the 15–19-year age group had the highest incidence and YLD rates, with AAPCs of -0.43 (95% CI, -0.51 to -0.36) and − 0.48 (-0.56 to -0.41), respectively. However, during this period, the < 5 years age group showed the lowest incidence and YLD rates. Overall, the number of incident cases and YLDs, as well as their rates, increased with age, while the incidence and YLD rates showed a descending trend between 1990 and 2019 across all age groups, accompanied by AAPCs below 0.

**Fig. 1:**
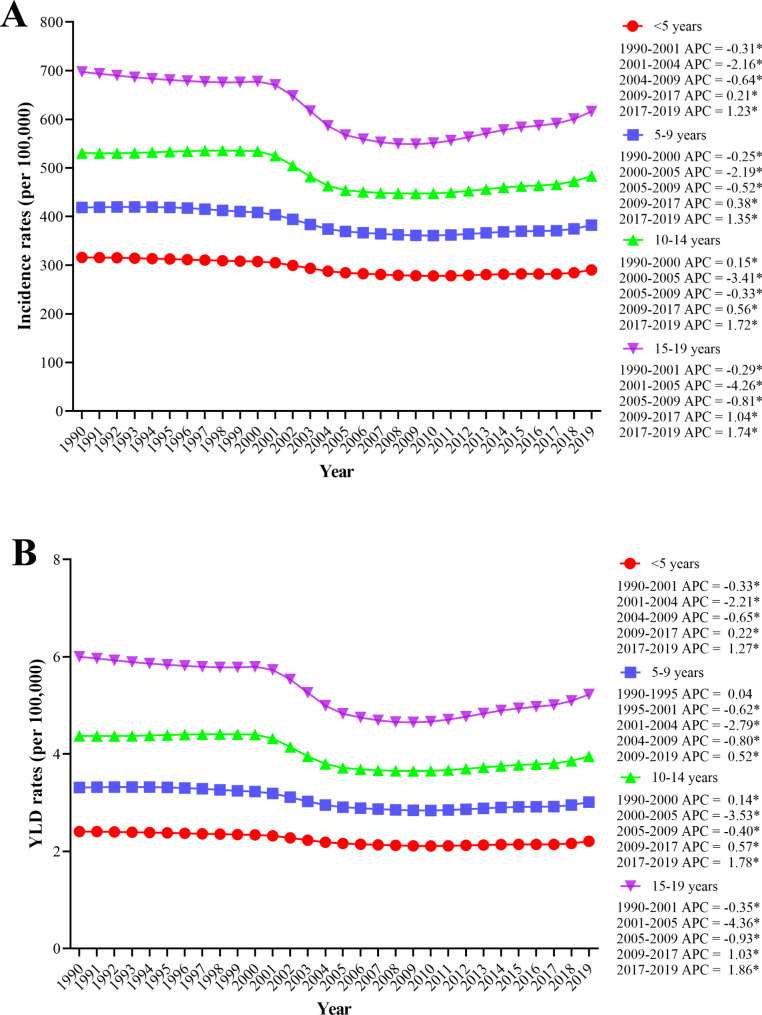
Joinpoint regression analysis of global incidence rates (**a**) and YLD rates (**b**) among 4 age groups in children and adolescents from 1990 to 2019. APC = annual percentage change; YLDs = years lived with disability. *P < 0.05

The joinpoint regression analysis indicated a downward trend of the global incidence rate of IOFB from 1990 to 2009 and a dramatic upward trend from 2009 to 2019 (Fig. 1A). For the 15–19 years age group, the decrease was most significant between 2001 and 2005, with an APC of -4.26, while the increase was most significant between 2017 and 2019, with an APC of 1.74. For the < 5 years age group, the incidence rates showed minimal decreasing trends between 2001 and 2004, with an APC of -2.16, and an increase between 2017 and 2019, with an APC of 1.23. The YLD rates of IOFBs (Fig. 1B) mostly showed a dramatic decline since 2001; however, they showed increasing trends from 2009 to 2019. Additionally, for all age groups, the 15–19 years age group showed the most obvious decreasing and increasing trend from 2001 to 2005 (APC: -4.36) and from 2017 to 2019 (APC: 1.8), respectively. The minimal trends were observed in the < 5 years age group; however, the YLD rates of other groups fluctuated. The joinpoint regression analysis of the global incidence rates showed significant temporal changes (*P* < 0.05).

### Sex-specific IOFB burden among children and adolescents

**Fig. 2:**
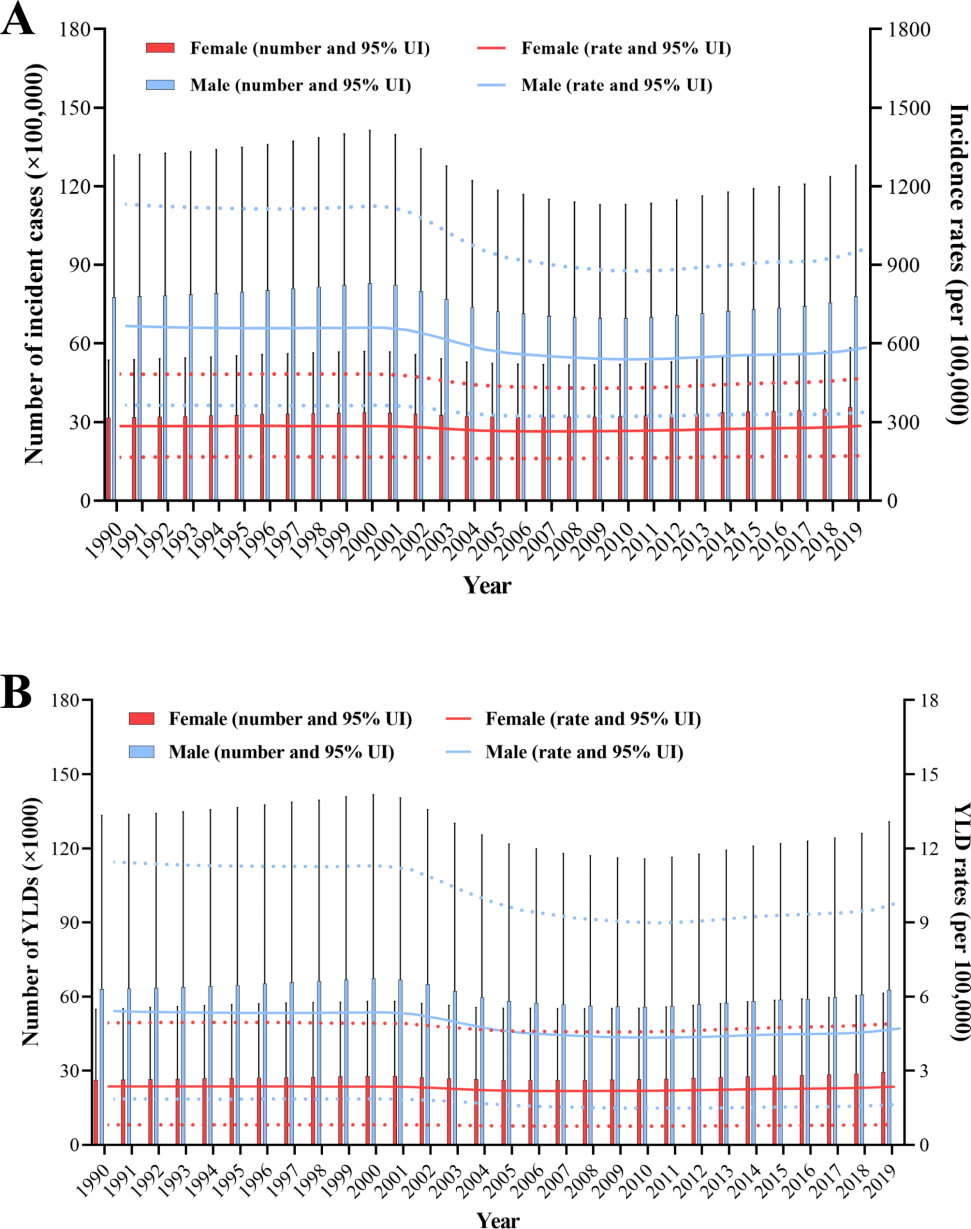
Global number of incident cases and incidence rates (**a**), and number of YLDs and YLD rates (**b**) for both females and males from 1990 to 2019. Red and blue dashed line represent the 95% UIs for females and males, respectively. YLDs = years lived with disability; UI = uncertainty interval

The number of cases and incidence rates were both lower among females [3.56 million (95% UI, 2.14–5.83) and 285.35 (95% UI, 171.41–466.40) per 100,000 population, respectively] than among males [7.78 million (95% UI, 4.50–12.80) and 585.37 (95%, UI 338.32–962.7) per 100,000 population, respectively] in 2019, respectively. These between-sex differences were larger in 1990 than in 2019. Among males, the incidence rates decreased between 1990 and 2019 [666.48 (95% UI, 364.53–1131.79) and 585.37 (95% UI, 338.32–926.7) per 100,000 population, respectively], with an AAPC of -0.44 (-0.53 to -0.36). For women, the AAPC of the incidence rates was 0. The YLDs among males significantly dropped, with an AAPC of -0.47, while those among females remained stable (Table 1). From 1990 to 2019, the number of incident cases and YLDs among males initially showed an upward trend, followed by a decrease, and a subsequent gradual increase to almost the initial level. However, the incidence and YLD rates among males started to decrease in the medium term and lasted until 2019. Contrastingly, the overall trends among females remained steady (Fig. 2A-B).

### Trends of IOFB burden according to region and country among children and adolescents

The incidence and YLD rates in most regions, countries, and territories increased from 1990 to 2019 (Table S1). Oceania had the lowest number of incident cases in 1990 (7140.81) and 2019 (13202.63), while East Asia and South Asia had the highest in 1990 (3.02 million) and 2019 (3.97 million), respectively. Central Asia and Oceania had the lowest incidence rates in 1990 (393.47 per 100,000 population) and 2019 (13202.63 per 100,000 population), respectively, while East Asia and tropical Latin America had the highest in 1990 (649.13 per 100,000 population) and 2019 (577.17 per 100,000 population), respectively. Although East Asia had the highest incidence rates in 1990, it showed the maximum decrease in incidence rates, with an AAPC of -1.58 (-2.06–1.1). Meanwhile, South-East Asia showed the maximum increase in incidence rates, with an AAPC of 0.17 (95% CI, 0.17–0.17). Contrastingly, East Asia and Tropical Latin America had the highest YLD rates in 1990 and 2019, respectively. On the contrary, Oceania had the lowest YLD rates in both 1990 and 2019. Furthermore, the AAPCs in both incidence and YLD rates decreased in high-income countries, except in Southern Latin America. For other high-income countries, although North America has the highest number of incident cases and YLDs, its incidence and YLD rates showed the fastest decrease.

**Fig. 3:**
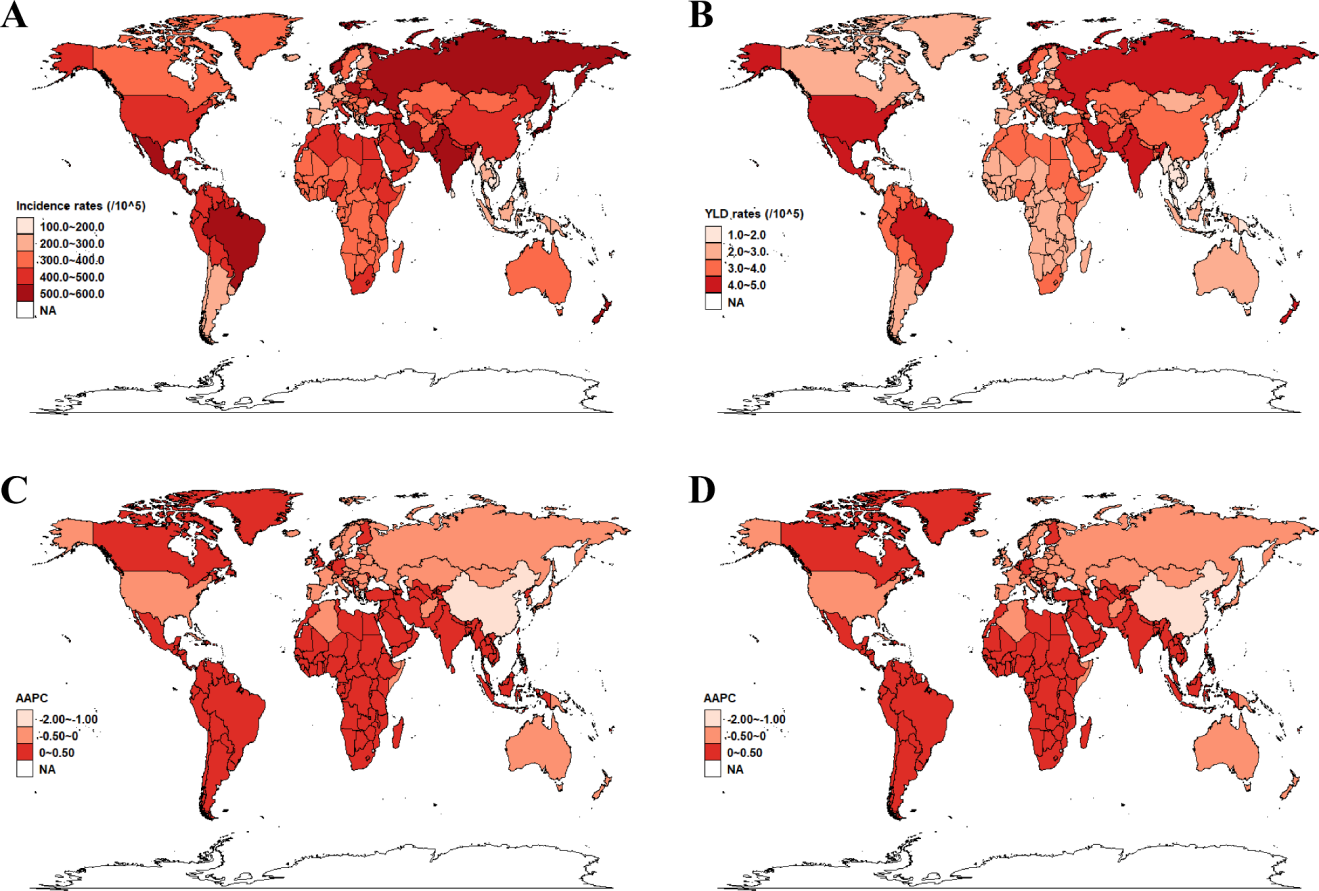
Global maps of incidence rates (**a**) and YLD rates (**b**) in 2019 as well as AAPCs in incidence (**c**) and YLD rates (**d**) from 1990 to 2019. AAPCs = average annual percentage changes; YLD = year lived with disability

In 2019, India (593.57 per 100,000 population), Brazil (583.73 per 100,000 population), and Mexico (582.15 per 100,000 population) had the highest incidence rates. In contrast, the Republic of Korea (157.07 per 100,000 population), Taiwan (159.49 per 100,000 population), and Myanmar (197.83 per 100,000 population) had the lowest incidence rates (Table S1, Fig. 3A). Notably, China showed the highest YLD rates in 1990 but it was replaced by Brazil in 2019. Mexico had higher YLD rates in both 1990 and 2019 (Fig. 3B). Overall, 154 countries and territories had AAPCs in incidence rates > 0, 9 had AAPCs that remained stable, and 41 had AAPCs < 0. From 1990 to 2019, the AAPCs in the incidence rates were highest in Equatorial Guinea (0.38), the Northern Mariana Islands (0.37), and the Syrian Arab Republic (0.3). Conversely, China (-1.60), Czechia (-0.15), and Georgia (-0.11) exhibited substantial descending trends (Table S1, Fig. 3C). Regarding YLD rates, a decreasing trend was observed in China (-1.61), while a dramatic increasing trend was observed in Northern Mariana Islands (0.41) (Table S1, Fig. 3D).

### Trends of IOFB burden by the SDI among children and adolescents

Generally, from 1990 to 2019, there were decreasing trends in the number of incident cases and YLDs in high, high-middle, and middle SDI regions, while there were increasing trends in low-middle and low SDI regions. In both 1990 and 2019, the highest number of incident cases and YLD were observed in moderate SDI regions, while the lowest numbers were observed in high SDI regions. Additionally, the middle and low-middle SDI regions presented the highest incidence rates in 1990 (521.42, 95% UI 262.32–938.70) and 2019 (478.28, 95% UI 287.22–765.23), respectively. These trends were also manifested in YLD rates. Countries in the high-middle and middle SDI regions showed reductions in the incidence and YLD rates from 1990 to 2019, while countries from low-middle and low SDI regions showed increase. High SDI regions maintained a stable incidence rate between 1990 and 2019. The largest decrease in the incidence and YLD rates were observed in the high-middle SDI regions, with AAPCs of -0.63 and − 0.66, respectively, while the largest increases were observed in the low-middle and low SDI regions, with AAPCs of 0.03 and 0.04, respectively (Table 1).

**Fig. 4:**
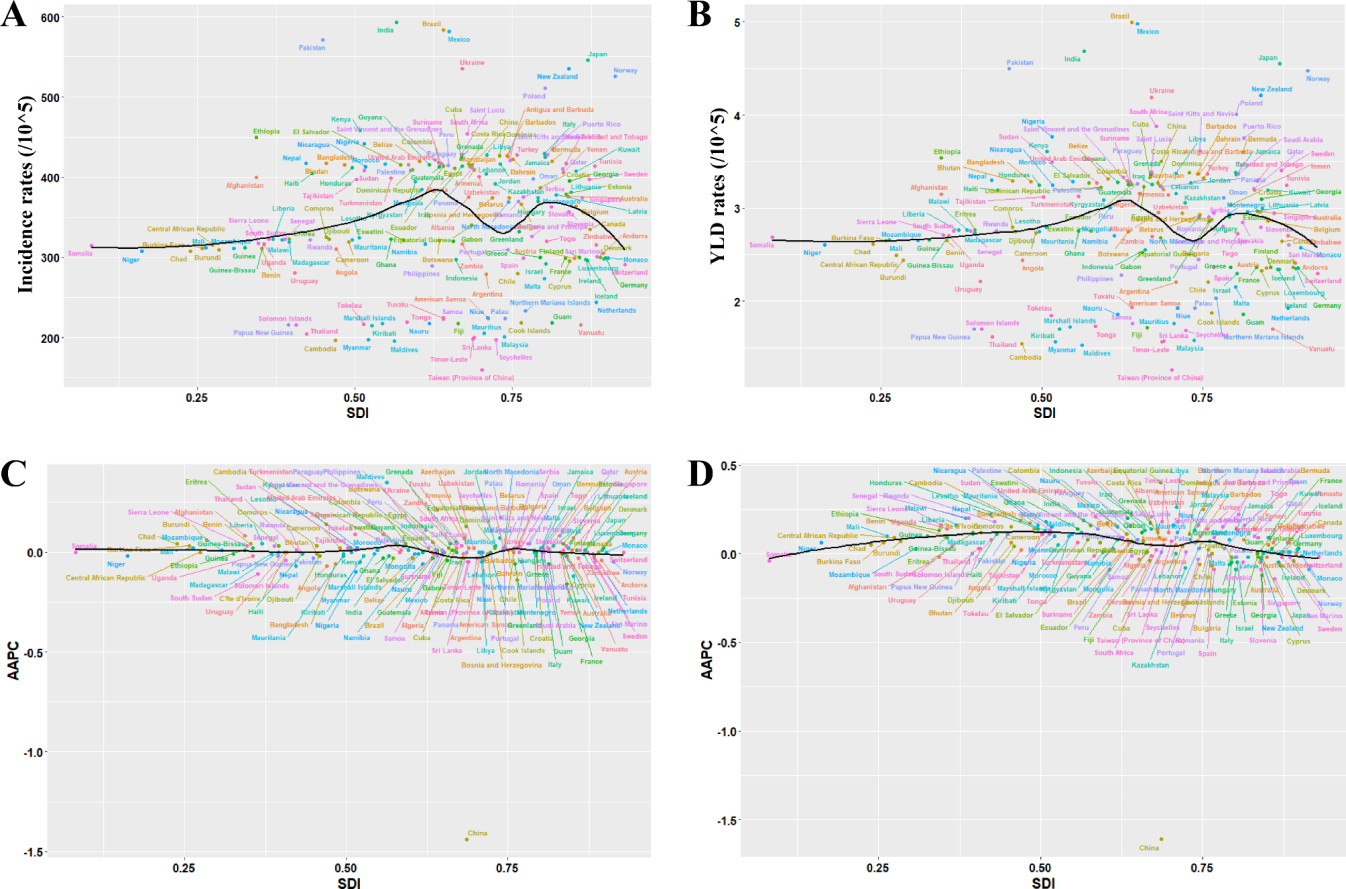
Global maps of incidence rates (**a**), and YLD rates (**b**) in 2019 as well as AAPCs in incidence rates (**c**), and YLD rates (**d**) from 1990 to 2019. The black line represents the expected values based on AAPCs and SDI in all locations. AAPCs = average annual percentage changes; YLD = year lived with disability; SDI = socio-demographic index

Overall, the trends in the incidence and YLD rates initially showed an increase at low SDI, peaking at the low-middle SDI, gradually decreasing at the middle SDI, and sharply decreasing at high-middle and high SDI (Fig. 4A and B), which was similar to the AAPCs in incidence and YLD rates observed between 1990 and 2019 (Fig. 4C and D). According to the SDI, India, Brazil, and Mexico showed with the highest incidence and YLD rates of IOFBs among children and adolescents (Fig. 4A and B). Notably, based on the SDI level, China had the lowest AAPC value in both the incidence and YLD rates between 1990 and 2019 (Fig. 4C and D).

## Discussion

This study evaluated the case numbers, incidence rates, and YLD rates from 1990 to 2019 based on sex, age, and across regional, national, and socioeconomic levels among children and adolescents. Overall, IOFBs in children and adolescents accounted for 11.35 million cases and 92,108 YLDs, with an incidence rate of 439.99 per 100,000 persons and an YLD rate of 3.57 per 100,000 persons in 2019. Between 1990 and 2019, the global incidence and YLD rates showed a downward trend (AAPC, -0.3 and − 0.32), whereas the number of incident cases and YLDs showed an upward trend that varied across age groups and regions. Therefore, our findings warrant the development of age- and region-based specific healthcare policies and may inform the development of early interventions.

Previous studies have demonstrated that IOFBs exhibit age-specific patterns in children and adolescents [16, 17]. The causes of IOFBs varied across the age groups. A > 10-year study on patients aged < 18 years with IOFBs in one of the most developed cities in China found that, among patients aged 0–3 years, the main cause of injury was uncertain (17.5%), followed by scissors (15.0%) and fireworks (15.0%). In the 4–12 years age group, fireworks accounted for the highest proportion of injuries (36.7%), followed by explosives (12.4%), plant branches, and pencil injuries (11%). Metal splashing (31.7%) was the most common cause of injury among children aged 13–17 years [2]. However, another study using the United States eye injury database reported less common causes of IOFB-related injuries such as assault, motor vehicle accidents, lawn mower, and firework injuries [5]. In present study, we found that individuals aged < 5 years had the lowest incidence and YLD rates of IOFBs between 1990 and 2019. This could be attributed to two reasons. On the one hand, young children were typically less able to identify the exact cause of IOFB and often had injuries due to uncertain causes. One the other hand, preschool children are given more attention and care from their families and society. Accordingly, a study conducted by the British Eye Surveillance Unit confirmed that most eye injuries in children in the UK occur in environments where direct supervision of affected children is expected (at their own homes, other homes, schools, or daycare centres), while very few such injuries occur in relatively unsupervised places (public places or sports facilities) [18]. Furthermore, since older children and adolescents have an expansive range of independent activities and opportunities to satisfy their curiosity, they are especially vulnerable. In our study, the incidence and YLD rates of IOFBs among older children and younger adolescents were moderate. However, the 15–19 years age group had the highest burden of number of cases, incidence rate, and YLD rate of IOFBs, which could be attributed to the similar clinical characteristics as those in adults with IOFBs, with 59.3% and 25.9% being related to work and daily activities, respectively [19]. In addition, they are more susceptible to IOFB injuries related to fireworks and metal splashes [20]. Most open eye injuries in children are caused by sharp penetrating objects such as knives, sharp metals, pencils/pens, sticks/wood, glass, and wires [21]. Notably, although the frequency of IOFBs increased with age, improvements in global health policies, such as the global initiative for the elimination of avoidable blindness by 2020 [19], decreased the burden of incidence and YLD rates within the past 30 years across all age groups.

We found that male children and adolescents accounted for most IOFB cases, with higher incidence and YLD rates. Similarly, GBD 2017 showed a higher burden of DALYs from blindness and vision loss in male children than in female children [22]. These findings are consistent with previous reports from the United States, which showed that the proportion of open globe injuries involving IOFBs was twice as high in boys than in girls, with the average age for all paediatric cases of open globe injury being 10.6 years [21]. In a Chinese study, males were more prone to IOFBs following exposure to fireworks and gunshot injuries, while plant branches were the primary cause of eye injuries in women [2]. Therefore, preventive measures for IOFB injuries, including safety education and effective safeguards, should be implemented according to sex. Notably, there has been a marked decrease in the incidence rates of IOFBs among males: from 666.48 per 100,000 population in 1990 to 585.37 per 100,000 population in 2019. Moreover, the burden of YLD rates also significantly declined, with an AAPC of -0.47. This could be attributed to following reasons, including the implementation of safer outdoor activity measures in developing countries such as fireworks management strategies and wearing safety glasses [23, 24]; reduced outdoor activity engagement among children and adolescents in high-income countries due to addictive electronic device use; and improved healthcare [25, 26]. However, there have been previous contrasting reports. In a rural community in Ethiopia, most people are injured during local unsafe food processing due to work, with women having a 1.16 times higher risk than men [27]. Therefore, safety education and protective measures for children and adolescents with IOFB injuries should take into account both gender and area.

Oceania, which is a region composed of Pacific Island countries, exhibited the lowest incidence and YLD rates of IOFBs in both 1990 and 2019, which may be attributed to the smaller population. East Asia in 1990 was replaced by South Asia in 2019 as the region with the highest number of cases. Specifically, as a member of South Asia, India exhibited the largest increase in the number of cases from 1990 (2.34 million) to 2019 (3.03 million) and the highest incidence rate at the country level, which was consistent with other GBD analyses [6]. This may reflect the lack of access to eye health care and health education India, and thus should be considered when planning eye care services [28]. Over the past 30 years, the number of cases in East Asia has decreased and the incidence rate has shown the most significant decrease, with an AAPC of -1.58. Specifically, China, which is the largest in terms of land and population in East Asia [29], has achieved a significant reduction in the number of IOFB cases from 1990 (3.00 million) to 2019 (1.25 million). This can be attributed to initiatives for improving awareness in children and adolescents and economic restructuring. In addition, there was a significant decreasing trend of YLD rate in China, with an AAPC of -1.61. To overcome population ageing and low fertility, China has made tremendous efforts to improve the health of children and adolescents by focusing on injury, risk behaviours, mental health, and vulnerability. Healthy China 2030 was proposed to prioritise health and ensure socioeconomic development, in accordance with the United Nations Sustainable Development Goals. Moreover, the Chinese government is committed to improving gender equality [30]. Therefore, these factors have resulted in a rapid decrease in male-dominant IOFB injuries among children and adolescents. One study indicated that between 2009 and 2019, only three countries showed downward or upward trends in the IOFB incidence rates, while the remaining countries exhibited volatile trends [31]. In contrast, we found that 154 countries and territories had stable AAPCs > 0 while 41 countries and territories had AAPCs < 0. In addition, Latin America, as the only high-income region with an increased AAPC, showed the highest incidence and YLD rate in 2019. Countries in Latin America, especially Brazil and Mexico, had the highest incidence and YLD rates in 2019. In these areas, funding cuts led to the interruption of social protection measures and food poverty has sharply intensified, which may lead to an increase in conflicts. Eyes are more vulnerable to injury during conflicts given their exposed anatomical positions, and the reduction of social security measures could impede those seeking medical treatment. Therefore, more social action, such as the “Vision for the Future” initiative in Brazil [32], and community education are needed to mitigate visual impairment in children and adolescents. Regarding war, since 2003, approximately 25% of the US military personnel seeking medical attention for eye injuries related to operations in Afghanistan and Iraq at the Walter Reed Army Medical Center Eye Service Center had IOFBs, with an average removal time of 21 days [33]. Delayed IOFB removal can lead to poor vision and an increased incidence of postoperative complications. Similarly, in another war-affected country, Syria, the average time from injury to IOFB removal is 12.2 ± 6.3 days, with the main cause of IOFBs being explosions. Notably, delayed IOFB removal in Syria did not result in a significant increase in serious eye complications [34].

Each geographical unit had an annual SDI score. Lower incidence and YLD rates were observed in the high, high-middle, and middle SDI regions, while higher incidence and YLD rates were observed in the low-middle and low SDI regions. Another study reported a clear substantial downward gradient in IOFB burden among children and adolescents with increasing SDI in 2019 [35]. Children and adolescents in low and low-middle SDI areas often suffer severe malnutrition; face difficulty in accessing potable water, sanitation facilities, and personal hygiene products; and are exposed to grave situations, such as war. Similarly, the Sustainable Development Goals initiative lacks focus on quantifying and minimising the disease burden on older children (> 5 years of age) and adolescents [36]. Thus, promoting socioeconomic development, improving healthcare facilities, and increasing public awareness in lower SDI areas are urgently required.

The current analysis of GBD data filled the gap regarding estimates of the IOFB burden among children and adolescents at the global, regional and national levels and highlighted the following points. First, for IOFBs in children and adolescents, the number of incident cases and YLDs increased from 1990 to 2019. Second, the number of incident cases, as well as the incidence and YLD rates, all increased with age; further, the causes of IOFBs differed according to age. Third, China, the most populous country in East Asia, demonstrated a noteworthy reduction in both the number of IOFB cases and YLD rates between 1990 and 2019. However, regional disparities were observed, particularly in the 15–18 age group, where male patients in low SDI regions experienced a significantly higher burden of IOFB disease. More importantly, over the past three decades, the gap between IOFB incidence rates and YLD rates has widened, particularly between high SDI regions and low SDI regions. This disparity underscores the unequal burden of IOFB-related disability, especially among male adolescents in low SDI areas, highlighting the urgent need for targeted interventions and healthcare resources allocation in these regions.

This study had some limitations. First, epidemiological data were absent or extremely limited in some regions, which might have increased measurement bias. Second, GBD 2019 did not identify the causes of IOFBs in children and adolescents. Third, resultant complications, including traumatic lens rupture, hyphaemia, vitreous haemorrhage, and endophthalmitis, may lead to underdiagnosis of IOFBs given the lack of a comprehensive definition for the same in the database. Fourth, the prevalence and DALYs due to IOFBs in children and adolescents were not examined. Lastly, it is essential to continually obtain information on IOFBs during childhood and adolescence beyond 2019 to improve our understanding of this important public health issue.

In summary, this study provided a comprehensive analysis of IOFBs among children and adolescents globally, highlighting significant trends and disparities. Our research results showed that from 1990 to 2019, there was a worrying increase in the number of cases and YLDs due to IOFBs, with reasons varying among different age groups. Gender-specific patterns indicated a higher burden in males, emphasizing the need for gender-sensitive preventive measures. Regional disparities, particularly in low SDI regions, emphasized the urgent requirement for improved healthcare access and public awareness. Despite these insights, limitations in data availability and pathogenic factors highlighted future research areas. Continued monitoring beyond 2019 is crucial for a more nuanced understanding of this public health challenge, providing targeted policies and interventions for the prevention and management of IOFB among children and adolescents worldwide.

### Electronic supplementary material

Below is the link to the electronic supplementary material.

Supplementary Material 1

## Data Availability

Publicly available datasets were analysed in this study. The data can be found here: https://vizhub.healthdata.org/gbd-results/.

## Abbreviations

AAPC: Average annual percentage changes
APC: Annual percent changes
CI: Confidence intervals
IOFB: Intraocular foreign bodies
OGI: Open-globe injury
SDI: Socio-demographic index
UI: Uncertainty interval
YLD: Years lived with disability
