# Supplementary material for: Global, regional, and national burdens of intraocular foreign bodies in children and adolescents from 1990 to 2019: a trend analysis
Source: BMC Public Health. 2023 Dec 12;23:2489. doi: 10.1186/s12889-023-17401-0 (PMC10717484; doi:10.1186/s12889-023-17401-0)
Supplement: Supplementary file 1 — Supplementary Material 1 [file 12889_2023_17401_MOESM1_ESM.doc]

**Table S1. Incidence and Years Lived with Disability of Intraocular Foreign Bodies and Their Average Annual Percentage Changes from 1990 to 2019 at the national Level**

|  | **Incidence** | | | | | **YLDs** | | | | |
| --- | --- | --- | --- | --- | --- | --- | --- | --- | --- | --- |
|  | **Number, 1990** | **Incidence rate**  **(per 100 000 Population), 1990** | **Number, 2019** | **Incidence rate**  **(per 100 000 Population), 2019** | **AAPC, 1990-2019** | **Number, 1990** | **YLD rate**  **(per 100 000 Population), 1990** | **Number, 2019** | **YLD rate**  **(per 100 000 Population), 2019** | **AAPC, 1990-2019** |
| Afghanistan | 25596 (16066-40444) | 400.29 (251.26-632.5) | 86420 (54405-137838) | 400.03 (251.84-638.04) | 0 (-0.03 - 0.02) | 202 (67-423) | 3.16 (1.05-6.61) | 681 (224-1434) | 3.15 (1.04-6.64) | -0.02 (-0.06 - 0.02) |
| Albania | 5246 (3193-8510) | 360.53 (219.42-584.89) | 2498 (1493-4103) | 375.19 (224.3-616.24) | 0.14 (0.12 - 0.16) | 41 (14-86) | 2.83 (0.94-5.9) | 20 (6-41) | 2.95 (0.97-6.17) | 0.14 (0.12 - 0.17) |
| Algeria | 54565 (34107-87046) | 403.9 (252.47-644.33) | 59518 (37385-94872) | 402.72 (252.96-641.94) | -0.01 (-0.03 - 0) | 430 (143-904) | 3.18 (1.06-6.69) | 466 (153-984) | 3.16 (1.03-6.66) | -0.03 (-0.05 - 0) |
| American Samoa | 51 (33-81) | 215 (137.7-339.62) | 51 (31-82) | 223.82 (138.16-362.11) | 0.14 (0.13 - 0.15) | 0 (0-1) | 1.83 (0.68-3.73) | 0 (0-1) | 1.93 (0.72-3.96) | 0.18 (0.16 - 0.2) |
| Andorra | 41 (25-67) | 305.24 (183.84-501.1) | 45 (27-75) | 304.76 (181.74-503.28) | 0 (-0.04 - 0.04) | 0 (0-1) | 2.42 (0.8-5.11) | 0 (0-1) | 2.41 (0.8-5.12) | 0 (-0.04 - 0.04) |
| Angola | 17856 (11334-28488) | 307.89 (195.43-491.23) | 52938 (33374-85628) | 310.38 (195.68-502.04) | 0.03 (0.02 - 0.03) | 141 (47-295) | 2.42 (0.81-5.09) | 416 (139-878) | 2.44 (0.81-5.15) | 0.02 (0.01 - 0.03) |
| Antigua and Barbuda | 100 (61-158) | 407.9 (250.74-648.02) | 101 (59-164) | 426.17 (249.5-689.62) | 0.15 (0.14 - 0.16) | 1 (0-2) | 3.21 (1.07-6.69) | 1 (0-2) | 3.36 (1.12-7.1) | 0.16 (0.15 - 0.18) |
| Argentina | 35801 (21981-58330) | 275.03 (168.86-448.1) | 39466 (24086-64263) | 278.86 (170.19-454.08) | 0.05 (0.04 - 0.05) | 283 (94-597) | 2.17 (0.72-4.58) | 312 (103-657) | 2.2 (0.73-4.64) | 0.05 (0.04 - 0.05) |
| Armenia | 5290 (3263-8477) | 398.81 (246-639.01) | 3171 (1948-5115) | 409.25 (251.41-660.23) | 0.09 (0.07 - 0.11) | 41 (14-87) | 3.13 (1.04-6.53) | 25 (8-52) | 3.2 (1.07-6.71) | 0.08 (0.06 - 0.11) |
| Australia | 19672 (12169-31678) | 380.24 (235.21-612.31) | 22644 (14063-36402) | 376.04 (233.53-604.52) | -0.04 (-0.05 - -0.03) | 155 (51-323) | 3 (0.98-6.24) | 178 (58-371) | 2.96 (0.97-6.17) | -0.05 (-0.06 - -0.03) |
| Austria | 5623 (3446-9134) | 300.1 (183.94-487.51) | 5209 (3204-8439) | 298.79 (183.77-484.08) | -0.02 (-0.03 - -0.01) | 44 (15-93) | 2.37 (0.78-4.98) | 41 (14-86) | 2.36 (0.78-4.95) | -0.02 (-0.04 - -0.01) |
| Azerbaijan | 12575 (7768-20094) | 401.27 (247.88-641.2) | 12371 (7569-19980) | 412.76 (252.53-666.64) | 0.1 (0.07 - 0.13) | 99 (33-207) | 3.15 (1.05-6.59) | 97 (32-204) | 3.24 (1.07-6.79) | 0.09 (0.06 - 0.12) |
| Bahamas | 446 (268-720) | 415.14 (249.67-670.26) | 469 (272-766) | 424.27 (246.49-692.73) | 0.07 (0.05 - 0.09) | 4 (1-7) | 3.28 (1.1-6.9) | 4 (1-8) | 3.35 (1.13-7.17) | 0.08 (0.05 - 0.1) |
| Bahrain | 794 (503-1285) | 395.08 (249.95-638.93) | 1355 (805-2187) | 422.33 (250.9-681.7) | 0.23 (0.17 - 0.3) | 6 (2-13) | 3.1 (1.01-6.49) | 11 (4-23) | 3.32 (1.11-7.07) | 0.25 (0.18 - 0.31) |
| Bangladesh | 238129 (150484-384495) | 396.69 (250.69-640.52) | 246751 (148062-397965) | 415.63 (249.4-670.34) | 0.16 (0.16 - 0.16) | 1876 (623-3942) | 3.13 (1.04-6.57) | 1950 (646-4106) | 3.28 (1.09-6.92) | 0.17 (0.16 - 0.18) |
| Barbados | 357 (213-575) | 418.79 (249.87-673.82) | 296 (173-480) | 427.21 (249.31-692.94) | 0.07 (0.05 - 0.08) | 3 (1-6) | 3.3 (1.11-6.97) | 2 (1-5) | 3.37 (1.13-7.14) | 0.07 (0.05 - 0.08) |
| Belarus | 11902 (7384-19184) | 377.85 (234.43-609.06) | 7812 (4895-12612) | 374.31 (234.52-604.29) | -0.03 (-0.08 - 0.01) | 93 (31-198) | 2.97 (0.99-6.28) | 61 (21-130) | 2.93 (0.98-6.21) | -0.06 (-0.1 - -0.01) |
| Belgium | 9047 (5628-14348) | 365.92 (227.61-580.3) | 9298 (5820-14969) | 363.42 (227.48-585.09) | 0.06 (-0.33 - 0.45) | 71 (24-148) | 2.89 (0.98-6) | 73 (25-153) | 2.86 (0.96-6) | 0.05 (-0.35 - 0.46) |
| Belize | 408 (255-651) | 401.45 (251.39-640.87) | 701 (416-1132) | 419.52 (248.73-676.79) | 0.15 (0.15 - 0.16) | 3 (1-7) | 3.16 (1.04-6.69) | 6 (2-12) | 3.31 (1.11-6.99) | 0.17 (0.16 - 0.18) |
| Benin | 8752 (5587-14034) | 304.7 (194.52-488.59) | 22113 (13948-35650) | 311.34 (196.38-501.93) | 0.07 (0.07 - 0.08) | 74 (27-150) | 2.58 (0.95-5.23) | 188 (69-384) | 2.65 (0.97-5.4) | 0.1 (0.09 - 0.11) |
| Bermuda | 66 (40-104) | 411.1 (250.24-654.18) | 50 (30-81) | 419.93 (249.33-676.85) | 0.08 (0.07 - 0.08) | 1 (0-1) | 3.51 (1.29-7.12) | 0 (0-1) | 3.6 (1.3-7.36) | 0.08 (0.07 - 0.09) |
| Bhutan | 1334 (832-2112) | 409.34 (255.09-647.84) | 1105 (662-1774) | 417.1 (249.93-669.75) | 0.07 (0.06 - 0.07) | 11 (4-22) | 3.24 (1.08-6.73) | 9 (3-18) | 3.29 (1.11-6.92) | 0.06 (0.05 - 0.07) |
| Bolivia (Plurinational State of) | 13431 (8480-21470) | 396.28 (250.21-633.48) | 20029 (12562-31763) | 400.96 (251.48-635.88) | 0.04 (0.03 - 0.05) | 106 (35-222) | 3.12 (1.05-6.56) | 157 (52-332) | 3.15 (1.04-6.64) | 0.03 (0.02 - 0.04) |
| Bosnia and Herzegovina | 5532 (3334-9024) | 369.33 (222.63-602.51) | 2487 (1485-4088) | 374.67 (223.77-615.9) | 0.05 (0.02 - 0.08) | 44 (14-91) | 2.91 (0.96-6.1) | 20 (6-41) | 2.95 (0.98-6.16) | 0.04 (0.01 - 0.07) |
| Botswana | 2263 (1395-3720) | 313.71 (193.4-515.58) | 2950 (1819-4792) | 321.16 (197.99-521.58) | 0.08 (0.07 - 0.09) | 19 (7-39) | 2.68 (0.98-5.43) | 25 (9-51) | 2.75 (1.01-5.57) | 0.08 (0.07 - 0.1) |
| Brazil | 386406 (233638-609578) | 571.94 (345.82-902.27) | 374747 (224462-588867) | 583.73 (349.64-917.26) | 0.07 (0.07 - 0.07) | 3307 (1213-6628) | 4.9 (1.8-9.81) | 3209 (1193-6523) | 5 (1.86-10.16) | 0.07 (0.07 - 0.08) |
| Brunei Darussalam | 424 (270-677) | 368.62 (234.16-587.94) | 504 (312-808) | 384.02 (237.43-614.7) | 0.14 (0.13 - 0.15) | 3 (1-7) | 2.9 (0.96-6) | 4 (1-8) | 3.03 (1.01-6.32) | 0.16 (0.15 - 0.16) |
| Bulgaria | 8787 (5259-14358) | 371.96 (222.64-607.83) | 4789 (2880-7832) | 366.8 (220.53-599.85) | -0.05 (-0.08 - -0.02) | 69 (23-145) | 2.93 (0.98-6.13) | 38 (12-79) | 2.88 (0.94-6.04) | -0.07 (-0.1 - -0.03) |
| Burkina Faso | 17408 (11039-28044) | 307.09 (194.74-494.72) | 39772 (25102-64142) | 311.44 (196.57-502.27) | 0.05 (0.05 - 0.05) | 148 (55-300) | 2.61 (0.96-5.29) | 338 (123-690) | 2.65 (0.96-5.4) | 0.06 (0.04 - 0.07) |
| Burundi | 9647 (6157-15359) | 304.06 (194.07-484.1) | 20631 (13016-33237) | 309.76 (195.43-499.03) | 0.06 (0.05 - 0.07) | 76 (26-159) | 2.39 (0.82-5.02) | 162 (54-342) | 2.44 (0.81-5.14) | 0.07 (0.05 - 0.09) |
| Cabo Verde | 603 (379-971) | 313.56 (197.28-505.38) | 681 (418-1122) | 325.82 (199.83-536.83) | 0.13 (0.12 - 0.15) | 5 (2-11) | 2.67 (0.97-5.46) | 6 (2-12) | 2.79 (1.03-5.64) | 0.14 (0.1 - 0.18) |
| Cambodia | 10754 (6700-17492) | 186.04 (115.9-302.59) | 12781 (7703-21102) | 196.16 (118.22-323.85) | 0.18 (0.17 - 0.2) | 85 (28-177) | 1.46 (0.49-3.07) | 101 (34-212) | 1.54 (0.51-3.26) | 0.19 (0.18 - 0.2) |
| Cameroon | 18183 (11577-29157) | 308.47 (196.4-494.63) | 48737 (30439-79532) | 319.55 (199.58-521.46) | 0.12 (0.12 - 0.13) | 155 (58-314) | 2.62 (0.98-5.32) | 417 (154-856) | 2.73 (1.01-5.61) | 0.14 (0.13 - 0.16) |
| Canada | 25648 (15844-40324) | 334.55 (206.67-525.98) | 27248 (16763-43221) | 335.12 (206.16-531.56) | 0.01 (0 - 0.02) | 202 (67-424) | 2.64 (0.87-5.53) | 215 (71-449) | 2.65 (0.88-5.52) | 0.01 (0 - 0.02) |
| Central African Republic | 4630 (2937-7406) | 308.56 (195.68-493.49) | 8890 (5584-14359) | 315.39 (198.1-509.41) | 0.08 (0.07 - 0.08) | 36 (12-77) | 2.43 (0.82-5.1) | 70 (23-147) | 2.49 (0.82-5.22) | 0.09 (0.08 - 0.09) |
| Chad | 10701 (6843-16961) | 303.85 (194.3-481.6) | 31056 (19744-49867) | 307.78 (195.67-494.21) | 0.04 (0.04 - 0.05) | 91 (33-185) | 2.58 (0.94-5.25) | 264 (97-537) | 2.61 (0.96-5.33) | 0.05 (0.04 - 0.06) |
| Chile | 14592 (9019-23517) | 276.43 (170.85-445.51) | 13603 (8273-22187) | 279.66 (170.09-456.13) | 0.03 (-0.01 - 0.07) | 115 (38-240) | 2.19 (0.72-4.56) | 107 (35-226) | 2.21 (0.72-4.65) | 0.02 (-0.07 - 0.11) |
| China | 2997454 (1024376-6125539) | 666.41 (227.74-1361.86) | 1247135 (423375-2607321) | 415.83 (141.17-869.36) | -1.6 (-2.07 - -1.12) | 25433 (8258-60320) | 5.65 (1.84-13.41) | 10500 (3363-24960) | 3.5 (1.12-8.32) | -1.61 (-2.14 - -1.08) |
| Colombia | 61137 (38046-97115) | 404.59 (251.78-642.68) | 62722 (37732-100623) | 417.85 (251.37-670.34) | 0.11 (0.11 - 0.12) | 482 (160-999) | 3.19 (1.06-6.61) | 494 (166-1040) | 3.29 (1.11-6.93) | 0.11 (0.1 - 0.12) |
| Comoros | 828 (522-1328) | 313.46 (197.49-502.72) | 992 (609-1634) | 325.5 (199.78-536.2) | 0.13 (0.13 - 0.13) | 7 (3-14) | 2.68 (0.99-5.46) | 9 (3-17) | 2.79 (1.03-5.7) | 0.15 (0.14 - 0.15) |
| Congo | 4276 (2695-6845) | 312.2 (196.76-499.81) | 7919 (4960-12875) | 316.95 (198.51-515.28) | 0.05 (0.04 - 0.06) | 34 (11-71) | 2.46 (0.82-5.16) | 62 (21-131) | 2.5 (0.83-5.26) | 0.05 (0.04 - 0.06) |
| Cook Islands | 19 (12-31) | 219.03 (138.17-352.55) | 12 (8-20) | 218.68 (136.04-353.36) | -0.01 (-0.03 - 0.02) | 0 (0-0) | 1.87 (0.7-3.82) | 0 (0-0) | 1.87 (0.69-3.86) | 0 (-0.03 - 0.03) |
| Costa Rica | 5706 (3579-9097) | 402.81 (252.67-642.2) | 5896 (3546-9500) | 416.17 (250.28-670.6) | 0.11 (0.1 - 0.12) | 45 (15-94) | 3.16 (1.04-6.65) | 46 (15-98) | 3.27 (1.08-6.88) | 0.12 (0.11 - 0.13) |
| Côte te d'Ivoire | 21359 (13586-34176) | 307.61 (195.66-492.18) | 42279 (26571-68042) | 317.29 (199.4-510.62) | 0.11 (0.1 - 0.11) | 181 (66-368) | 2.61 (0.96-5.3) | 360 (134-735) | 2.7 (1.01-5.52) | 0.12 (0.11 - 0.13) |
| Croatia | 5174 (3152-8331) | 390.13 (237.7-628.22) | 3180 (1956-5104) | 386.92 (237.98-621.09) | -0.02 (-0.05 - 0.01) | 41 (13-86) | 3.07 (1.02-6.45) | 25 (8-53) | 3.04 (1-6.4) | -0.02 (-0.05 - 0) |
| Cuba | 15489 (9236-24942) | 425.87 (253.95-685.78) | 10482 (6263-16893) | 422.81 (252.62-681.43) | -0.02 (-0.04 - -0.01) | 122 (42-258) | 3.37 (1.15-7.08) | 82 (28-174) | 3.33 (1.12-7.01) | -0.04 (-0.05 - -0.02) |
| Cyprus | 768 (473-1256) | 296.66 (182.45-484.86) | 823 (511-1334) | 295.51 (183.6-478.8) | -0.01 (-0.02 - -0.01) | 6 (2-13) | 2.34 (0.77-4.93) | 6 (2-14) | 2.33 (0.76-4.9) | -0.02 (-0.03 - -0.01) |
| Czechia | 10819 (6517-17015) | 355.71 (214.27-559.44) | 7417 (4548-11873) | 340.58 (208.85-545.19) | -0.15 (-0.18 - -0.12) | 85 (28-182) | 2.8 (0.94-5.98) | 58 (19-123) | 2.67 (0.88-5.64) | -0.17 (-0.21 - -0.14) |
| Democratic People's Republic of Korea | 12592 (8072-19162) | 150.69 (96.6-229.33) | 10530 (6458-16410) | 157.07 (96.33-244.78) | 0.14 (0.14 - 0.15) | 99 (33-205) | 1.19 (0.4-2.46) | 83 (28-177) | 1.24 (0.42-2.64) | 0.17 (0.16 - 0.17) |
| Democratic Republic of the Congo | 67825 (43258-108008) | 307.17 (195.91-489.15) | 149717 (93905-242467) | 316.65 (198.61-512.82) | 0.1 (0.1 - 0.11) | 533 (178-1119) | 2.42 (0.8-5.07) | 1180 (391-2479) | 2.5 (0.83-5.24) | 0.11 (0.1 - 0.12) |
| Denmark | 3780 (2300-6181) | 302.38 (183.97-494.41) | 3903 (2384-6353) | 299.51 (182.9-487.43) | -0.03 (-0.04 - -0.02) | 30 (10-63) | 2.39 (0.8-5.04) | 31 (10-65) | 2.36 (0.78-4.98) | -0.04 (-0.05 - -0.03) |
| Djibouti | 878 (554-1400) | 319.29 (201.73-509.32) | 1695 (1064-2733) | 323.04 (202.76-520.83) | 0.04 (0.03 - 0.05) | 7 (3-15) | 2.72 (1-5.52) | 14 (5-30) | 2.75 (0.99-5.65) | 0.04 (0.02 - 0.05) |
| Dominica | 135 (82-216) | 415.64 (252.46-665.51) | 86 (50-140) | 427.28 (248.98-693.97) | 0.1 (0.09 - 0.1) | 1 (0-2) | 3.28 (1.11-6.86) | 1 (0-1) | 3.38 (1.13-7.14) | 0.11 (0.1 - 0.12) |
| Dominican Republic | 14264 (8898-22629) | 401.65 (250.57-637.21) | 16447 (10090-26091) | 409.71 (251.35-649.95) | 0.07 (0.06 - 0.08) | 113 (37-232) | 3.17 (1.04-6.54) | 130 (43-269) | 3.23 (1.07-6.7) | 0.06 (0.05 - 0.07) |
| Ecuador | 20218 (12613-32139) | 404.36 (252.25-642.76) | 27651 (16766-44264) | 413.49 (250.71-661.92) | 0.08 (0.07 - 0.08) | 160 (53-332) | 3.19 (1.06-6.64) | 218 (73-454) | 3.26 (1.09-6.79) | 0.07 (0.06 - 0.08) |
| Egypt | 112371 (70627-178180) | 401.75 (252.51-637.03) | 172247 (107003-274650) | 410.96 (255.3-655.29) | 0.08 (0.07 - 0.09) | 885 (296-1870) | 3.16 (1.06-6.69) | 1354 (456-2821) | 3.23 (1.09-6.73) | 0.07 (0.06 - 0.08) |
| El Salvador | 11034 (6849-17517) | 405.56 (251.75-643.86) | 9521 (5752-15277) | 415.46 (250.99-666.62) | 0.08 (0.07 - 0.1) | 87 (29-181) | 3.2 (1.06-6.66) | 75 (25-158) | 3.27 (1.1-6.88) | 0.08 (0.06 - 0.1) |
| Equatorial Guinea | 754 (484-1193) | 304.58 (195.27-481.77) | 2538 (1552-4187) | 339.88 (207.81-560.77) | 0.38 (0.38 - 0.39) | 6 (2-12) | 2.39 (0.8-5.01) | 20 (7-42) | 2.68 (0.88-5.62) | 0.4 (0.39 - 0.4) |
| Eritrea | 5436 (3428-8749) | 313.21 (197.5-504.11) | 10963 (6811-17928) | 322.26 (200.22-527) | 0.1 (0.09 - 0.11) | 46 (17-94) | 2.67 (0.99-5.44) | 94 (35-191) | 2.77 (1.03-5.63) | 0.12 (0.11 - 0.13) |
| Estonia | 1744 (1079-2794) | 379.64 (234.84-608.03) | 1049 (653-1701) | 378.87 (235.78-614.05) | -0.01 (-0.05 - 0.03) | 14 (5-29) | 2.98 (0.99-6.31) | 8 (3-17) | 2.97 (0.99-6.3) | -0.02 (-0.06 - 0.02) |
| Eswatini | 1470 (919-2393) | 308.38 (192.69-501.94) | 1709 (1057-2778) | 321.78 (198.97-523.1) | 0.15 (0.14 - 0.15) | 13 (5-26) | 2.63 (0.96-5.36) | 15 (5-30) | 2.76 (1.02-5.58) | 0.16 (0.15 - 0.18) |
| Ethiopia | 129891 (80914-203607) | 433.44 (270.01-679.43) | 263632 (160637-416809) | 449.73 (274.03-711.03) | 0.13 (0.12 - 0.13) | 1021 (341-2112) | 3.41 (1.14-7.05) | 2075 (689-4300) | 3.54 (1.18-7.33) | 0.13 (0.13 - 0.14) |
| Fiji | 778 (490-1257) | 216.59 (136.4-349.84) | 744 (467-1201) | 217.51 (136.56-351.4) | 0.01 (0.01 - 0.02) | 6 (2-13) | 1.71 (0.57-3.62) | 6 (2-12) | 1.72 (0.57-3.63) | 0.01 (0 - 0.02) |
| Finland | 3771 (2335-6164) | 297.04 (183.96-485.61) | 3511 (2157-5780) | 299.79 (184.17-493.59) | 0.04 (-0.01 - 0.08) | 30 (10-63) | 2.34 (0.78-4.94) | 28 (9-58) | 2.37 (0.79-4.94) | 0.03 (0 - 0.07) |
| France | 48342 (29629-78578) | 299.59 (183.61-486.96) | 47683 (29023-77895) | 299.32 (182.19-488.97) | 0 (-0.01 - 0) | 382 (127-801) | 2.37 (0.78-4.97) | 376 (123-794) | 2.36 (0.77-4.98) | 0 (-0.01 - 0) |
| Gabon | 1608 (1015-2582) | 309.72 (195.51-497.24) | 2435 (1506-3998) | 320.35 (198.11-525.89) | 0.12 (0.11 - 0.12) | 13 (4-27) | 2.44 (0.81-5.13) | 19 (6-40) | 2.53 (0.82-5.29) | 0.12 (0.12 - 0.13) |
| Gambia | 1750 (1117-2792) | 306.15 (195.42-488.56) | 3777 (2358-6159) | 319.01 (199.14-520.16) | 0.14 (0.14 - 0.15) | 15 (5-30) | 2.6 (0.94-5.31) | 32 (12-66) | 2.73 (1.02-5.54) | 0.17 (0.16 - 0.18) |
| Georgia | 7308 (4442-11770) | 407.21 (247.55-655.85) | 3643 (2315-5796) | 395.19 (251.17-628.76) | -0.11 (-0.15 - -0.08) | 57 (19-120) | 3.2 (1.06-6.67) | 29 (10-59) | 3.1 (1.04-6.38) | -0.12 (-0.16 - -0.08) |
| Germany | 51502 (31805-83555) | 297.04 (183.44-481.91) | 46979 (28826-76204) | 299.11 (183.53-485.19) | 0.03 (0.02 - 0.04) | 406 (134-857) | 2.34 (0.77-4.94) | 371 (122-781) | 2.36 (0.78-4.97) | 0.03 (0.02 - 0.04) |
| Ghana | 25856 (16315-41711) | 311.73 (196.7-502.89) | 47046 (29204-77002) | 322.35 (200.1-527.61) | 0.12 (0.11 - 0.12) | 220 (80-450) | 2.65 (0.96-5.43) | 403 (147-823) | 2.76 (1-5.64) | 0.14 (0.13 - 0.14) |
| Greece | 8525 (5124-13991) | 303.33 (182.3-497.81) | 5909 (3595-9636) | 299.59 (182.28-488.57) | -0.05 (-0.06 - -0.04) | 67 (22-143) | 2.4 (0.79-5.09) | 47 (15-98) | 2.37 (0.78-4.99) | -0.05 (-0.07 - -0.04) |
| Greenland | 59 (37-91) | 328.16 (208.94-507.72) | 51 (32-79) | 332.46 (207.13-521.2) | 0.05 (0.02 - 0.07) | 1 (0-1) | 2.81 (1.05-5.7) | 0 (0-1) | 2.86 (1.07-5.77) | 0.07 (0.03 - 0.1) |
| Grenada | 166 (103-265) | 406.53 (252.36-649.94) | 132 (77-213) | 425.53 (250.34-687.6) | 0.16 (0.15 - 0.17) | 1 (0-3) | 3.2 (1.06-6.69) | 1 (0-2) | 3.36 (1.12-7.12) | 0.17 (0.16 - 0.19) |
| Guam | 118 (75-187) | 217.16 (137.68-344.6) | 128 (81-206) | 218.11 (137.62-351.19) | 0.01 (0 - 0.03) | 1 (0-2) | 1.85 (0.7-3.75) | 1 (0-2) | 1.86 (0.69-3.79) | 0.02 (0 - 0.04) |
| Guatemala | 17459 (11088-28053) | 391.68 (248.76-629.36) | 31917 (19401-51027) | 413.06 (251.08-660.38) | 0.19 (0.17 - 0.2) | 137 (45-287) | 3.08 (1.02-6.45) | 252 (84-529) | 3.26 (1.09-6.84) | 0.2 (0.18 - 0.21) |
| Guinea | 10381 (6641-16455) | 304.72 (194.93-483.01) | 21977 (13857-35458) | 312.22 (196.87-503.74) | 0.08 (0.08 - 0.09) | 88 (32-180) | 2.58 (0.95-5.27) | 187 (68-384) | 2.66 (0.96-5.45) | 0.1 (0.09 - 0.11) |
| Guinea-Bissau | 1802 (1139-2903) | 309.24 (195.45-498.29) | 3173 (1990-5138) | 316.87 (198.8-513.21) | 0.08 (0.08 - 0.09) | 15 (6-31) | 2.64 (0.97-5.36) | 27 (10-55) | 2.71 (0.99-5.52) | 0.09 (0.08 - 0.11) |
| Guyana | 1511 (928-2396) | 406.21 (249.51-644.1) | 1170 (706-1879) | 415.4 (250.62-667.17) | 0.08 (0.07 - 0.09) | 12 (4-25) | 3.21 (1.07-6.67) | 9 (3-19) | 3.28 (1.11-6.86) | 0.08 (0.07 - 0.09) |
| Haiti | 13031 (8240-20911) | 391.92 (247.81-628.92) | 22268 (13841-35376) | 404.77 (251.6-643.04) | 0.11 (0.1 - 0.12) | 103 (35-217) | 3.09 (1.04-6.52) | 176 (58-365) | 3.19 (1.06-6.63) | 0.11 (0.1 - 0.12) |
| Honduras | 10743 (6764-17368) | 396.27 (249.52-640.65) | 17973 (10941-28707) | 411.96 (250.77-657.99) | 0.13 (0.13 - 0.14) | 85 (28-178) | 3.12 (1.02-6.58) | 142 (47-296) | 3.25 (1.09-6.79) | 0.14 (0.14 - 0.15) |
| Hungary | 10886 (6468-17916) | 375.5 (223.12-618) | 6925 (4156-11307) | 370.26 (222.23-604.58) | -0.05 (-0.06 - -0.04) | 86 (29-180) | 2.96 (0.99-6.2) | 54 (18-114) | 2.91 (0.96-6.11) | -0.06 (-0.07 - -0.05) |
| Iceland | 251 (155-408) | 296.89 (182.76-482.01) | 263 (161-429) | 296.79 (181.61-485.04) | 0 (-0.02 - 0.01) | 2 (1-4) | 2.34 (0.78-4.96) | 2 (1-4) | 2.34 (0.76-4.97) | 0 (-0.02 - 0.01) |
| India | 2335145 (1431497-3645011) | 563.04 (345.16-878.88) | 3030202 (1806695-4778851) | 593.57 (353.9-936.1) | 0.18 (0.18 - 0.19) | 18394 (6008-38334) | 4.44 (1.45-9.24) | 23940 (7810-49836) | 4.69 (1.53-9.76) | 0.19 (0.19 - 0.2) |
| Indonesia | 252550 (152829-421571) | 287.48 (173.97-479.88) | 261940 (157133-433850) | 297.64 (178.55-492.98) | 0.12 (0.11 - 0.13) | 2160 (798-4340) | 2.46 (0.91-4.94) | 2248 (840-4541) | 2.55 (0.95-5.16) | 0.13 (0.12 - 0.14) |
| Iran (Islamic Republic of) | 178926 (109726-279440) | 557.97 (342.17-871.41) | 146590 (89169-229958) | 566.87 (344.82-889.25) | 0.06 (0.03 - 0.09) | 1403 (461-2945) | 4.38 (1.44-9.18) | 1148 (374-2393) | 4.44 (1.44-9.25) | 0.05 (0.02 - 0.09) |
| Iraq | 39830 (25148-63894) | 397.29 (250.84-637.32) | 75241 (45871-120260) | 413.63 (252.17-661.12) | 0.14 (0.12 - 0.15) | 313 (103-657) | 3.12 (1.03-6.55) | 592 (197-1240) | 3.25 (1.08-6.82) | 0.14 (0.13 - 0.16) |
| Ireland | 3990 (2415-6536) | 300.44 (181.89-492.14) | 3976 (2431-6515) | 296.86 (181.51-486.51) | -0.04 (-0.05 - -0.04) | 32 (10-66) | 2.37 (0.77-5.01) | 31 (10-66) | 2.34 (0.77-4.96) | -0.05 (-0.06 - -0.04) |
| Israel | 5933 (3678-9653) | 295.85 (183.41-481.31) | 9689 (6083-15612) | 292.38 (183.57-471.12) | -0.04 (-0.05 - -0.03) | 47 (15-99) | 2.33 (0.76-4.92) | 76 (25-160) | 2.3 (0.75-4.81) | -0.05 (-0.06 - -0.04) |
| Italy | 59650 (35316-96363) | 437.95 (259.3-707.5) | 46498 (27638-75503) | 429.06 (255.04-696.71) | -0.07 (-0.08 - -0.06) | 472 (158-998) | 3.47 (1.16-7.33) | 367 (121-771) | 3.38 (1.12-7.11) | -0.09 (-0.09 - -0.08) |
| Jamaica | 4470 (2733-7141) | 409.79 (250.59-654.7) | 3672 (2153-5953) | 425.18 (249.23-689.26) | 0.13 (0.12 - 0.14) | 35 (12-74) | 3.23 (1.07-6.75) | 29 (10-62) | 3.36 (1.13-7.12) | 0.14 (0.13 - 0.15) |
| Japan | 185362 (113295-295722) | 555.78 (339.7-886.67) | 117927 (72826-188651) | 546.03 (337.2-873.5) | -0.06 (-0.07 - -0.06) | 1550 (552-3227) | 4.65 (1.66-9.68) | 983 (352-2023) | 4.55 (1.63-9.37) | -0.07 (-0.08 - -0.06) |
| Jordan | 8670 (5413-13760) | 406.57 (253.82-645.26) | 20452 (12385-32898) | 416.42 (252.18-669.84) | 0.08 (0.08 - 0.09) | 68 (23-142) | 3.2 (1.06-6.64) | 161 (54-339) | 3.27 (1.1-6.9) | 0.08 (0.06 - 0.09) |
| Kazakhstan | 26388 (16241-42321) | 398.86 (245.49-639.7) | 24527 (15289-39516) | 391.66 (244.13-630.99) | -0.06 (-0.08 - -0.03) | 207 (69-434) | 3.13 (1.04-6.55) | 192 (63-400) | 3.06 (1-6.39) | -0.08 (-0.11 - -0.05) |
| Kenya | 60355 (37475-94858) | 435.98 (270.7-685.22) | 114615 (68879-182891) | 458.03 (275.26-730.88) | 0.17 (0.16 - 0.18) | 474 (158-981) | 3.43 (1.14-7.09) | 904 (299-1868) | 3.61 (1.2-7.47) | 0.18 (0.17 - 0.19) |
| Kiribati | 77 (49-122) | 212.2 (136.05-335.3) | 114 (72-183) | 214.85 (136.41-345.43) | 0.04 (0.03 - 0.05) | 1 (0-1) | 1.67 (0.56-3.5) | 1 (0-2) | 1.7 (0.56-3.6) | 0.05 (0.03 - 0.07) |
| Kuwait | 2774 (1744-4483) | 397.13 (249.71-641.8) | 4466 (2790-7129) | 407.33 (254.43-650.21) | 0.09 (0.06 - 0.11) | 22 (7-46) | 3.11 (1.02-6.56) | 35 (12-73) | 3.19 (1.06-6.7) | 0.09 (0.07 - 0.11) |
| Kyrgyzstan | 8232 (5101-13215) | 392.26 (243.07-629.66) | 10306 (6401-16681) | 394.29 (244.91-638.21) | 0.02 (0 - 0.04) | 65 (21-135) | 3.08 (1.01-6.43) | 81 (26-170) | 3.09 (1.01-6.49) | 0.01 (-0.02 - 0.04) |
| Lao People's Democratic Republic | 4241 (2624-6972) | 188.33 (116.53-309.64) | 5803 (3494-9504) | 196.26 (118.15-321.42) | 0.14 (0.14 - 0.15) | 33 (11-70) | 1.48 (0.5-3.13) | 46 (15-96) | 1.55 (0.52-3.26) | 0.15 (0.14 - 0.15) |
| Latvia | 2849 (1760-4551) | 378.67 (233.84-604.75) | 1483 (921-2388) | 378.13 (234.99-609.15) | -0.01 (-0.04 - 0.02) | 22 (8-47) | 2.98 (1-6.29) | 12 (4-25) | 2.96 (0.99-6.28) | -0.02 (-0.06 - 0.01) |
| Lebanon | 6270 (3942-9948) | 402.47 (253.08-638.58) | 6975 (4386-11153) | 403.73 (253.89-645.54) | 0.01 (-0.01 - 0.03) | 49 (16-104) | 3.16 (1.04-6.64) | 55 (18-115) | 3.16 (1.04-6.67) | 0 (-0.02 - 0.02) |
| Lesotho | 3017 (1867-4941) | 314.33 (194.53-514.72) | 2855 (1734-4601) | 324.25 (196.92-522.49) | 0.11 (0.1 - 0.12) | 26 (10-52) | 2.69 (0.99-5.44) | 25 (9-50) | 2.79 (1.04-5.65) | 0.13 (0.11 - 0.15) |
| Liberia | 3419 (2153-5523) | 309.88 (195.16-500.62) | 7733 (4784-12704) | 322.46 (199.5-529.75) | 0.13 (0.12 - 0.15) | 29 (11-59) | 2.64 (0.96-5.37) | 66 (25-135) | 2.76 (1.04-5.63) | 0.16 (0.14 - 0.18) |
| Libya | 9419 (5917-14969) | 398.91 (250.59-634) | 8804 (5122-14310) | 428.13 (249.09-695.92) | 0.24 (0.23 - 0.26) | 74 (24-155) | 3.13 (1.04-6.57) | 69 (23-148) | 3.38 (1.14-7.18) | 0.26 (0.25 - 0.28) |
| Lithuania | 4212 (2600-6738) | 380.89 (235.13-609.33) | 2115 (1306-3384) | 381.76 (235.72-610.78) | 0.01 (-0.03 - 0.05) | 33 (11-70) | 2.99 (1.01-6.33) | 17 (6-35) | 2.99 (1.02-6.33) | 0 (-0.05 - 0.05) |
| Luxembourg | 262 (162-425) | 296.73 (183.45-481.43) | 394 (242-642) | 298.1 (182.56-485.26) | 0.02 (0 - 0.03) | 2 (1-4) | 2.34 (0.76-4.92) | 3 (1-7) | 2.35 (0.77-4.98) | 0.02 (0 - 0.03) |
| Madagascar | 21020 (13274-33832) | 310.29 (195.95-499.41) | 44242 (27727-71937) | 318.94 (199.88-518.59) | 0.1 (0.09 - 0.1) | 179 (66-366) | 2.64 (0.97-5.4) | 379 (138-777) | 2.73 (0.99-5.6) | 0.11 (0.1 - 0.12) |
| Malawi | 16669 (10545-26711) | 307.39 (194.46-492.58) | 32761 (20155-54075) | 322 (198.1-531.5) | 0.16 (0.15 - 0.18) | 142 (52-288) | 2.61 (0.95-5.32) | 281 (104-574) | 2.77 (1.02-5.64) | 0.2 (0.18 - 0.22) |
| Malaysia | 16128 (9803-26613) | 193.29 (117.49-318.96) | 20923 (12425-34154) | 200.77 (119.22-327.73) | 0.13 (0.12 - 0.15) | 127 (42-266) | 1.52 (0.5-3.19) | 165 (55-350) | 1.58 (0.52-3.36) | 0.13 (0.12 - 0.15) |
| Maldives | 240 (149-392) | 187.88 (116.43-306.2) | 274 (167-450) | 195.35 (119-321.12) | 0.14 (0.11 - 0.16) | 2 (1-4) | 1.47 (0.49-3.09) | 2 (1-5) | 1.53 (0.5-3.22) | 0.12 (0.1 - 0.14) |
| Mali | 15048 (9626-23922) | 304.78 (194.97-484.51) | 39868 (25141-64098) | 312.06 (196.79-501.72) | 0.08 (0.08 - 0.09) | 127 (48-259) | 2.58 (0.97-5.25) | 339 (126-695) | 2.66 (0.99-5.44) | 0.1 (0.09 - 0.11) |
| Malta | 316 (194-505) | 274.48 (168.58-438.22) | 228 (141-363) | 273.61 (169.36-434.48) | -0.01 (-0.03 - 0.01) | 2 (1-5) | 2.17 (0.72-4.58) | 2 (1-4) | 2.16 (0.72-4.57) | -0.02 (-0.05 - 0.01) |
| Marshall Islands | 57 (37-93) | 212.65 (135.04-343.17) | 52 (33-84) | 217.8 (136.26-352.35) | 0.08 (0.08 - 0.09) | 0 (0-1) | 1.68 (0.56-3.55) | 0 (0-1) | 1.72 (0.57-3.63) | 0.1 (0.08 - 0.11) |
| Mauritania | 3541 (2247-5676) | 309.23 (196.18-495.66) | 6629 (4097-10923) | 321.44 (198.67-529.66) | 0.13 (0.13 - 0.14) | 30 (11-62) | 2.63 (0.96-5.39) | 57 (21-116) | 2.75 (1.01-5.63) | 0.16 (0.14 - 0.17) |
| Mauritius | 853 (505-1405) | 197.64 (116.86-325.34) | 633 (371-1040) | 205.27 (120.5-337.47) | 0.14 (0.11 - 0.17) | 7 (3-15) | 1.69 (0.62-3.46) | 5 (2-11) | 1.76 (0.66-3.62) | 0.16 (0.12 - 0.2) |
| Mexico | 246767 (149419-386431) | 568.03 (343.95-889.52) | 251593 (150816-395684) | 582.15 (348.97-915.56) | 0.08 (0.08 - 0.09) | 2112 (783-4221) | 4.86 (1.8-9.72) | 2154 (788-4355) | 4.98 (1.82-10.08) | 0.08 (0.08 - 0.09) |
| Micronesia (Federated States of) | 126 (80-202) | 214.78 (136.61-344.57) | 95 (59-153) | 222.9 (137.65-360.55) | 0.13 (0.12 - 0.13) | 1 (0-2) | 1.7 (0.55-3.61) | 1 (0-2) | 1.77 (0.58-3.72) | 0.14 (0.13 - 0.15) |
| Monaco | 15 (9-24) | 299.99 (183.71-488.36) | 20 (12-33) | 299.05 (182.79-487.5) | -0.01 (-0.03 - 0.01) | 0 (0-0) | 2.59 (0.95-5.26) | 0 (0-0) | 2.58 (0.96-5.24) | -0.01 (-0.03 - 0.01) |
| Mongolia | 4450 (2760-7117) | 392.89 (243.69-628.4) | 4734 (2966-7578) | 384.5 (240.88-615.52) | -0.07 (-0.11 - -0.04) | 35 (12-73) | 3.09 (1.03-6.46) | 37 (12-77) | 3.01 (0.99-6.26) | -0.09 (-0.13 - -0.06) |
| Montenegro | 785 (475-1280) | 365.97 (221.18-596.34) | 560 (336-917) | 373.97 (224.34-612.51) | 0.07 (0.07 - 0.08) | 6 (2-13) | 2.87 (0.94-6.02) | 4 (1-9) | 2.94 (0.98-6.16) | 0.08 (0.07 - 0.09) |
| Morocco | 50344 (31498-80045) | 402.94 (252.1-640.66) | 52197 (31486-84029) | 416.13 (251.02-669.9) | 0.11 (0.11 - 0.11) | 397 (131-827) | 3.18 (1.05-6.62) | 411 (138-867) | 3.28 (1.1-6.91) | 0.11 (0.1 - 0.12) |
| Mozambique | 22922 (14502-37042) | 308.64 (195.26-498.77) | 52972 (33380-85426) | 311.36 (196.2-502.12) | 0.03 (0.02 - 0.03) | 195 (72-398) | 2.63 (0.97-5.35) | 451 (167-923) | 2.65 (0.98-5.42) | 0.03 (0.02 - 0.04) |
| Myanmar | 38307 (23318-63105) | 192.13 (116.96-316.52) | 39446 (23392-64535) | 197.83 (117.31-323.65) | 0.1 (0.1 - 0.1) | 302 (100-632) | 1.52 (0.5-3.17) | 311 (103-661) | 1.56 (0.52-3.31) | 0.1 (0.1 - 0.11) |
| Namibia | 2368 (1474-3863) | 313.3 (195-511.07) | 3411 (2114-5552) | 316.41 (196.14-515.13) | 0.03 (0.03 - 0.04) | 20 (7-41) | 2.68 (0.99-5.44) | 29 (11-59) | 2.71 (1-5.47) | 0.03 (0.02 - 0.04) |
| Nauru | 11 (7-18) | 209.63 (135.57-329.16) | 11 (7-18) | 217.53 (137.79-349.18) | 0.13 (0.12 - 0.13) | 0 (0-0) | 1.78 (0.65-3.6) | 0 (0-0) | 1.86 (0.69-3.75) | 0.15 (0.14 - 0.16) |
| Nepal | 41026 (26032-65934) | 393.58 (249.73-632.52) | 52275 (31385-84010) | 417.56 (250.69-671.05) | 0.2 (0.2 - 0.21) | 323 (107-676) | 3.1 (1.02-6.48) | 414 (139-877) | 3.3 (1.11-7) | 0.22 (0.21 - 0.23) |
| Netherlands | 9342 (5674-15070) | 243.75 (148.04-393.21) | 9183 (5542-14986) | 243.6 (147.01-397.51) | 0.01 (-0.13 - 0.16) | 74 (24-154) | 1.93 (0.64-4.01) | 73 (24-152) | 1.92 (0.63-4.02) | 0.01 (-0.13 - 0.15) |
| New Zealand | 5938 (3694-9430) | 539.2 (335.44-856.3) | 6341 (3929-10139) | 535.45 (331.75-856.16) | -0.02 (-0.03 - -0.02) | 47 (16-98) | 4.25 (1.45-8.93) | 50 (17-105) | 4.21 (1.43-8.89) | -0.03 (-0.04 - -0.02) |
| Nicaragua | 8934 (5622-14412) | 396.34 (249.4-639.39) | 10863 (6623-17395) | 413.22 (251.93-661.69) | 0.14 (0.14 - 0.15) | 70 (23-148) | 3.11 (1.03-6.59) | 85 (29-178) | 3.25 (1.08-6.78) | 0.15 (0.14 - 0.15) |
| Niger | 14651 (9371-23410) | 302.6 (193.54-483.5) | 44744 (28437-71745) | 307.35 (195.34-492.83) | 0.05 (0.04 - 0.06) | 124 (45-252) | 2.57 (0.94-5.21) | 380 (141-772) | 2.61 (0.97-5.3) | 0.06 (0.04 - 0.07) |
| Nigeria | 208342 (130036-325300) | 427.39 (266.76-667.32) | 520215 (318407-824879) | 441.58 (270.28-700.2) | 0.11 (0.1 - 0.12) | 1772 (650-3563) | 3.64 (1.33-7.31) | 4434 (1625-8883) | 3.76 (1.38-7.54) | 0.12 (0.11 - 0.13) |
| Niue | 2 (1-4) | 218.24 (138.03-351.98) | 1 (1-2) | 224.71 (138.93-363.92) | 0.1 (0.09 - 0.11) | 0 (0-0) | 1.86 (0.67-3.77) | 0 (0-0) | 1.93 (0.71-3.99) | 0.12 (0.1 - 0.14) |
| North Macedonia | 2584 (1562-4210) | 365.18 (220.73-594.93) | 1726 (1042-2815) | 369.26 (222.88-602.05) | 0.04 (0.01 - 0.06) | 20 (7-43) | 2.87 (0.94-6.01) | 14 (4-28) | 2.9 (0.95-6.07) | 0.03 (0 - 0.06) |
| Northern Mariana Islands | 35 (22-55) | 211.59 (133.84-332.41) | 27 (16-44) | 233.34 (138.66-378.15) | 0.37 (0.24 - 0.49) | 0 (0-1) | 1.81 (0.67-3.65) | 0 (0-0) | 2.03 (0.76-4.19) | 0.41 (0.33 - 0.49) |
| Norway | 5996 (3605-9858) | 538.55 (323.74-885.38) | 6601 (3968-10941) | 525.76 (316.05-871.41) | -0.08 (-0.1 - -0.07) | 51 (19-104) | 4.6 (1.69-9.31) | 56 (21-114) | 4.48 (1.64-9.1) | -0.09 (-0.11 - -0.08) |
| Oman | 3919 (2464-6325) | 394.15 (247.83-636.14) | 5110 (3217-8229) | 398.34 (250.76-641.53) | 0.04 (-0.04 - 0.13) | 31 (10-64) | 3.09 (1.02-6.48) | 40 (13-85) | 3.11 (1.02-6.6) | 0.03 (-0.03 - 0.09) |
| Pakistan | 346435 (214381-536503) | 553.89 (342.76-857.77) | 638908 (387705-1001335) | 571.13 (346.58-895.11) | 0.11 (0.1 - 0.11) | 2727 (887-5709) | 4.36 (1.42-9.13) | 5035 (1666-10517) | 4.5 (1.49-9.4) | 0.11 (0.1 - 0.11) |
| Palau | 14 (9-22) | 220.57 (137.87-352.27) | 10 (6-17) | 223.94 (137.54-364.81) | 0.05 (0.04 - 0.07) | 0 (0-0) | 1.89 (0.7-3.84) | 0 (0-0) | 1.93 (0.73-3.97) | 0.06 (0.04 - 0.09) |
| Palestine | 4801 (3055-7668) | 393.36 (250.27-628.3) | 9705 (6037-15475) | 408.64 (254.2-651.6) | 0.13 (0.13 - 0.14) | 38 (12-78) | 3.08 (1.01-6.42) | 76 (25-159) | 3.21 (1.05-6.69) | 0.14 (0.13 - 0.14) |
| Panama | 4453 (2741-7080) | 409.76 (252.2-651.47) | 6148 (3775-9801) | 411.57 (252.68-656.08) | 0.02 (0.01 - 0.02) | 35 (12-73) | 3.23 (1.07-6.74) | 48 (16-101) | 3.23 (1.07-6.75) | 0.01 (0 - 0.01) |
| Papua New Guinea | 4547 (2908-7226) | 215.59 (137.89-342.66) | 10000 (6405-15811) | 215.73 (138.17-341.08) | 0 (0 - 0.01) | 36 (12-76) | 1.7 (0.57-3.59) | 79 (26-166) | 1.7 (0.57-3.58) | 0 (-0.01 - 0.01) |
| Paraguay | 8251 (5193-13296) | 398.01 (250.51-641.43) | 10886 (6576-17513) | 416.21 (251.43-669.57) | 0.15 (0.15 - 0.16) | 65 (21-137) | 3.13 (1.04-6.6) | 86 (29-180) | 3.28 (1.09-6.88) | 0.16 (0.16 - 0.17) |
| Peru | 43423 (27121-69018) | 404.07 (252.37-642.23) | 49755 (30409-79456) | 414.69 (253.44-662.24) | 0.09 (0.09 - 0.09) | 342 (113-713) | 3.19 (1.05-6.64) | 391 (130-815) | 3.26 (1.09-6.79) | 0.08 (0.07 - 0.09) |
| Philippines | 91164 (55742-150326) | 282.46 (172.71-465.77) | 134161 (81776-213666) | 289.71 (176.59-461.4) | 0.09 (0.09 - 0.09) | 717 (242-1473) | 2.22 (0.75-4.56) | 1055 (352-2222) | 2.28 (0.76-4.8) | 0.09 (0.08 - 0.09) |
| Poland | 63629 (38031-103421) | 512.04 (306.05-832.27) | 39388 (23712-63428) | 511.01 (307.63-822.88) | -0.01 (-0.04 - 0.01) | 500 (167-1043) | 4.02 (1.34-8.39) | 309 (102-645) | 4 (1.33-8.37) | -0.02 (-0.05 - 0.01) |
| Portugal | 9276 (5573-15031) | 311.31 (187.02-504.46) | 6099 (3745-9719) | 309.55 (190.07-493.3) | -0.02 (-0.03 - -0.01) | 74 (25-153) | 2.47 (0.83-5.13) | 48 (16-102) | 2.45 (0.81-5.15) | -0.03 (-0.04 - -0.02) |
| Puerto Rico | 5556 (3346-8944) | 417.03 (251.1-671.26) | 3281 (1888-5351) | 434.84 (250.18-709.12) | 0.14 (0.14 - 0.15) | 48 (18-98) | 3.58 (1.33-7.35) | 28 (11-58) | 3.75 (1.39-7.63) | 0.16 (0.15 - 0.17) |
| Qatar | 609 (384-978) | 398.5 (251.6-640.57) | 2124 (1321-3392) | 410.32 (255.09-655.16) | 0.1 (0.09 - 0.12) | 5 (2-10) | 3.12 (1.04-6.55) | 17 (5-35) | 3.21 (1.05-6.81) | 0.1 (0.08 - 0.11) |
| Republic of Korea | 61725 (37675-99462) | 385.87 (235.52-621.78) | 36618 (22548-58701) | 384.14 (236.54-615.81) | -0.02 (-0.03 - 0) | 489 (164-1015) | 3.06 (1.02-6.35) | 289 (95-601) | 3.03 (1-6.31) | -0.03 (-0.05 - -0.01) |
| Republic of Moldova | 5893 (3690-9494) | 373.94 (234.12-602.41) | 2998 (1834-4872) | 387.91 (237.24-630.39) | 0.13 (0.09 - 0.17) | 46 (15-98) | 2.94 (0.98-6.19) | 24 (8-50) | 3.05 (1.02-6.53) | 0.13 (0.09 - 0.17) |
| Romania | 27759 (16644-45319) | 369.87 (221.76-603.85) | 15024 (8987-24550) | 370.28 (221.49-605.04) | 0 (-0.03 - 0.03) | 219 (72-457) | 2.92 (0.96-6.09) | 118 (39-248) | 2.91 (0.96-6.1) | -0.01 (-0.05 - 0.03) |
| Russian Federation | 239716 (146748-394444) | 529.72 (324.28-871.64) | 180287 (110872-298592) | 524.37 (322.47-868.46) | -0.04 (-0.08 - 0) | 1879 (623-3964) | 4.15 (1.38-8.76) | 1408 (465-2993) | 4.1 (1.35-8.7) | -0.05 (-0.1 - -0.01) |
| Rwanda | 12675 (8055-20355) | 307.23 (195.25-493.39) | 20099 (12427-33022) | 321.57 (198.83-528.33) | 0.16 (0.13 - 0.18) | 108 (40-220) | 2.62 (0.96-5.34) | 173 (63-351) | 2.76 (1.01-5.61) | 0.18 (0.17 - 0.2) |
| Saint Kitts and Nevis | 75 (46-120) | 410.34 (251.33-655.68) | 68 (40-111) | 421.01 (248.41-681.09) | 0.09 (0.08 - 0.09) | 1 (0-1) | 3.51 (1.3-7.21) | 1 (0-1) | 3.62 (1.33-7.36) | 0.1 (0.09 - 0.11) |
| Saint Lucia | 274 (170-435) | 405.22 (250.93-643.66) | 194 (113-314) | 428.95 (250.23-694.45) | 0.2 (0.19 - 0.2) | 2 (1-5) | 3.19 (1.07-6.67) | 2 (1-3) | 3.39 (1.15-7.12) | 0.21 (0.2 - 0.22) |
| Saint Vincent and the Grenadines | 223 (136-358) | 411.77 (251.26-660.64) | 144 (85-233) | 421.56 (250.03-680.98) | 0.08 (0.08 - 0.09) | 2 (1-4) | 3.25 (1.08-6.78) | 1 (0-2) | 3.33 (1.13-7.04) | 0.09 (0.08 - 0.09) |
| Samoa | 192 (119-314) | 223.34 (138.14-364.49) | 217 (133-356) | 222.46 (136.44-365.01) | -0.01 (-0.02 - -0.01) | 2 (1-3) | 1.77 (0.59-3.74) | 2 (1-4) | 1.76 (0.58-3.7) | -0.01 (-0.02 - -0.01) |
| San Marino | 20 (12-32) | 301.9 (183.48-494.35) | 22 (14-36) | 301.02 (182.83-491.6) | -0.01 (-0.02 - -0.01) | 0 (0-0) | 2.62 (0.99-5.34) | 0 (0-0) | 2.6 (0.96-5.3) | -0.02 (-0.03 - -0.01) |
| Sao Tome and Principe | 220 (139-356) | 314.44 (198.09-508.03) | 317 (193-526) | 326.27 (198.83-540.78) | 0.13 (0.12 - 0.13) | 2 (1-4) | 2.68 (0.99-5.48) | 3 (1-6) | 2.8 (1.05-5.71) | 0.15 (0.14 - 0.16) |
| Saudi Arabia | 33513 (21064-53466) | 401.66 (252.45-640.8) | 41355 (24577-66667) | 427.58 (254.11-689.3) | 0.22 (0.2 - 0.23) | 263 (88-557) | 3.16 (1.05-6.68) | 325 (108-689) | 3.36 (1.12-7.12) | 0.22 (0.2 - 0.24) |
| Senegal | 13596 (8655-21836) | 306.51 (195.13-492.29) | 25100 (15635-40969) | 322.7 (201.01-526.71) | 0.18 (0.17 - 0.18) | 116 (42-234) | 2.61 (0.94-5.29) | 215 (79-435) | 2.76 (1.01-5.6) | 0.2 (0.18 - 0.22) |
| Serbia | 10256 (6139-16772) | 364.48 (218.18-596.06) | 7462 (4471-12218) | 374.79 (224.59-613.65) | 0.1 (0.05 - 0.14) | 81 (27-169) | 2.87 (0.96-6.02) | 59 (19-123) | 2.95 (0.97-6.19) | 0.09 (0.05 - 0.12) |
| Seychelles | 62 (37-101) | 196.33 (117.67-322.51) | 57 (34-93) | 197.46 (118.21-323.38) | 0.02 (0.01 - 0.03) | 1 (0-1) | 1.68 (0.61-3.44) | 0 (0-1) | 1.68 (0.62-3.46) | 0.01 (0 - 0.02) |
| Sierra Leone | 5934 (3777-9494) | 305.69 (194.6-489.11) | 13549 (8490-22041) | 317.34 (198.86-516.24) | 0.13 (0.12 - 0.15) | 50 (19-103) | 2.6 (0.97-5.32) | 116 (43-236) | 2.71 (1.01-5.53) | 0.16 (0.14 - 0.17) |
| Singapore | 3573 (2182-5682) | 387.95 (236.95-616.97) | 3874 (2444-6209) | 374 (235.92-599.38) | -0.13 (-0.17 - -0.09) | 28 (9-58) | 3.07 (1.02-6.34) | 30 (10-63) | 2.94 (0.96-6.08) | -0.14 (-0.19 - -0.1) |
| Slovakia | 6445 (3861-10550) | 367.32 (220.03-601.28) | 4046 (2451-6583) | 363.21 (220-590.96) | -0.05 (-0.06 - -0.03) | 51 (17-106) | 2.89 (0.96-6.06) | 32 (11-67) | 2.85 (0.94-5.98) | -0.05 (-0.07 - -0.04) |
| Slovenia | 2033 (1183-3277) | 365.26 (212.59-588.74) | 1437 (853-2288) | 356.67 (211.75-568.02) | -0.09 (-0.11 - -0.06) | 16 (5-34) | 2.87 (0.94-6.05) | 11 (4-24) | 2.79 (0.91-5.89) | -0.09 (-0.11 - -0.07) |
| Solomon Islands | 420 (269-665) | 213.96 (137.07-339.11) | 703 (449-1122) | 215.86 (137.81-344.41) | 0.03 (0.03 - 0.03) | 3 (1-7) | 1.69 (0.56-3.56) | 6 (2-12) | 1.7 (0.57-3.58) | 0.03 (0.02 - 0.03) |
| Somalia | 13581 (8559-21924) | 318.96 (201.02-514.9) | 37195 (23437-59696) | 315.22 (198.62-505.91) | -0.04 (-0.05 - -0.03) | 116 (43-237) | 2.72 (1.01-5.58) | 317 (117-647) | 2.69 (0.99-5.48) | -0.04 (-0.06 - -0.02) |
| South Africa | 77698 (48134-128735) | 449.93 (278.73-745.47) | 88893 (54972-147540) | 453.9 (280.69-753.35) | 0.03 (0.02 - 0.04) | 665 (240-1341) | 3.85 (1.39-7.76) | 760 (275-1532) | 3.88 (1.4-7.82) | 0.02 (0.01 - 0.03) |
| South Sudan | 10441 (6560-16791) | 318.71 (200.25-512.51) | 17071 (10675-27885) | 322.33 (201.56-526.52) | 0.04 (0.03 - 0.05) | 89 (33-182) | 2.72 (0.99-5.56) | 146 (54-298) | 2.76 (1.02-5.62) | 0.04 (0.03 - 0.05) |
| Spain | 34090 (20402-56139) | 305.86 (183.05-503.68) | 27066 (16409-44335) | 299.44 (181.54-490.51) | -0.08 (-0.09 - -0.06) | 270 (89-574) | 2.42 (0.79-5.15) | 214 (70-451) | 2.36 (0.78-4.99) | -0.09 (-0.1 - -0.08) |
| Sri Lanka | 14381 (8576-23834) | 196.64 (117.26-325.9) | 13645 (8016-22475) | 199.89 (117.44-329.25) | 0.06 (0.05 - 0.07) | 113 (38-241) | 1.55 (0.52-3.3) | 107 (36-229) | 1.57 (0.52-3.35) | 0.05 (0.04 - 0.06) |
| Sudan | 44389 (28150-70786) | 395.36 (250.73-630.48) | 82789 (51472-131419) | 408.77 (254.14-648.89) | 0.12 (0.11 - 0.12) | 378 (138-769) | 3.36 (1.23-6.85) | 707 (257-1446) | 3.49 (1.27-7.14) | 0.13 (0.12 - 0.14) |
| Suriname | 698 (430-1114) | 409.68 (252.17-653.36) | 807 (488-1302) | 416.81 (252.02-672.34) | 0.06 (0.06 - 0.07) | 6 (2-12) | 3.23 (1.07-6.75) | 6 (2-13) | 3.29 (1.09-6.9) | 0.06 (0.05 - 0.07) |
| Sweden | 8400 (5224-13529) | 398.61 (247.9-641.96) | 9411 (5892-15381) | 397.12 (248.61-649.01) | 0 (-0.06 - 0.06) | 72 (27-147) | 3.43 (1.26-6.97) | 81 (30-166) | 3.41 (1.27-7.02) | -0.02 (-0.12 - 0.09) |
| Switzerland | 4613 (2849-7342) | 292.8 (180.83-466) | 5074 (3149-8149) | 291.28 (180.76-467.82) | -0.02 (-0.03 - 0) | 36 (12-76) | 2.31 (0.75-4.81) | 40 (13-83) | 2.29 (0.76-4.78) | -0.02 (-0.04 - -0.01) |
| Syrian Arab Republic | 29956 (18906-48121) | 398.31 (251.39-639.85) | 25522 (14793-41267) | 433.67 (251.37-701.2) | 0.3 (0.29 - 0.3) | 235 (78-496) | 3.13 (1.04-6.59) | 202 (68-426) | 3.43 (1.15-7.24) | 0.32 (0.31 - 0.33) |
| Taiwan (Province of China) | 11442 (6979-18328) | 156.38 (95.39-250.48) | 6811 (4146-10581) | 159.49 (97.09-247.77) | 0.07 (0.06 - 0.08) | 91 (31-191) | 1.24 (0.42-2.61) | 54 (18-113) | 1.26 (0.42-2.65) | 0.07 (0.05 - 0.08) |
| Tajikistan | 11049 (6914-17599) | 385.27 (241.08-613.69) | 16352 (10108-26218) | 397.08 (245.46-636.67) | 0.1 (0.09 - 0.12) | 87 (29-181) | 3.02 (1-6.32) | 128 (42-269) | 3.12 (1.03-6.54) | 0.11 (0.09 - 0.13) |
| Thailand | 45740 (26944-75184) | 199.82 (117.71-328.45) | 30645 (18001-50519) | 204.88 (120.34-337.75) | 0.09 (0.07 - 0.1) | 361 (119-768) | 1.58 (0.52-3.36) | 242 (80-515) | 1.62 (0.53-3.45) | 0.08 (0.07 - 0.09) |
| Timor-Leste | 773 (484-1244) | 189.18 (118.55-304.53) | 1317 (790-2150) | 198.09 (118.82-323.29) | 0.16 (0.16 - 0.16) | 6 (2-13) | 1.49 (0.49-3.1) | 10 (3-22) | 1.56 (0.52-3.31) | 0.18 (0.17 - 0.19) |
| Togo | 6710 (4246-10826) | 309.54 (195.89-499.46) | 12653 (7926-20556) | 319.81 (200.34-519.56) | 0.11 (0.11 - 0.11) | 57 (21-116) | 2.63 (0.97-5.35) | 108 (40-219) | 2.73 (1-5.54) | 0.13 (0.12 - 0.14) |
| Tokelau | 2 (1-3) | 210.43 (135.64-330.68) | 1 (1-2) | 216.82 (138.36-345.32) | 0.11 (0.09 - 0.12) | 0 (0-0) | 1.79 (0.65-3.61) | 0 (0-0) | 1.84 (0.68-3.74) | 0.11 (0.09 - 0.13) |
| Tonga | 112 (70-182) | 219.07 (137.29-354.27) | 102 (64-164) | 218.92 (137.43-353.28) | 0 (0 - 0) | 1 (0-2) | 1.73 (0.58-3.66) | 1 (0-2) | 1.73 (0.57-3.64) | -0.01 (-0.02 - 0) |
| Trinidad and Tobago | 2109 (1307-3376) | 407.73 (252.64-652.73) | 1509 (907-2438) | 417.32 (250.82-674.27) | 0.08 (0.05 - 0.11) | 17 (5-35) | 3.21 (1.06-6.73) | 12 (4-25) | 3.29 (1.1-6.96) | 0.09 (0.05 - 0.12) |
| Tunisia | 16544 (10335-26309) | 406.07 (253.67-645.75) | 14295 (8764-22848) | 414.11 (253.9-661.91) | 0.07 (0.05 - 0.09) | 130 (44-270) | 3.2 (1.07-6.63) | 112 (37-234) | 3.25 (1.08-6.79) | 0.06 (0.04 - 0.08) |
| Turkey | 114836 (70703-182317) | 409.85 (252.34-650.7) | 95275 (55930-153699) | 427.35 (250.87-689.4) | 0.14 (0.13 - 0.15) | 906 (301-1890) | 3.23 (1.07-6.75) | 751 (252-1579) | 3.37 (1.13-7.08) | 0.14 (0.13 - 0.15) |
| Turkmenistan | 7380 (4597-11787) | 390.95 (243.51-624.46) | 7649 (4727-12300) | 399.26 (246.73-642.04) | 0.07 (0.05 - 0.1) | 58 (19-121) | 3.07 (1.01-6.4) | 60 (20-125) | 3.13 (1.03-6.55) | 0.06 (0.04 - 0.09) |
| Tuvalu | 9 (6-14) | 212.69 (137.02-334.94) | 10 (6-17) | 224.25 (138.86-362.1) | 0.18 (0.18 - 0.19) | 0 (0-0) | 1.81 (0.66-3.67) | 0 (0-0) | 1.93 (0.71-3.95) | 0.22 (0.22 - 0.23) |
| Uganda | 31556 (20156-50101) | 305.89 (195.38-485.64) | 75746 (47590-122306) | 316.69 (198.97-511.36) | 0.12 (0.11 - 0.13) | 268 (97-546) | 2.6 (0.94-5.3) | 647 (235-1326) | 2.7 (0.98-5.55) | 0.14 (0.13 - 0.15) |
| Ukraine | 80391 (48925-132113) | 535.27 (325.76-879.65) | 47988 (29018-79735) | 535.63 (323.9-889.99) | 0 (-0.03 - 0.03) | 631 (210-1325) | 4.2 (1.4-8.83) | 376 (125-789) | 4.19 (1.4-8.81) | -0.01 (-0.04 - 0.03) |
| United Arab Emirates | 2784 (1762-4458) | 392.5 (248.47-628.51) | 6299 (3844-10152) | 415.57 (253.61-669.83) | 0.2 (0.09 - 0.3) | 22 (7-46) | 3.07 (1-6.47) | 50 (16-104) | 3.27 (1.08-6.87) | 0.21 (0.1 - 0.32) |
| United Kingdom | 59678 (36107-95666) | 400.72 (242.45-642.37) | 62419 (37722-100357) | 401.71 (242.77-645.86) | 0.01 (-0.01 - 0.02) | 476 (160-987) | 3.19 (1.08-6.63) | 496 (166-1042) | 3.19 (1.07-6.71) | 0 (-0.02 - 0.01) |
| United Republic of Tanzania | 46394 (29304-74546) | 309.59 (195.55-497.45) | 96183 (60524-154511) | 313.32 (197.16-503.32) | 0.04 (0.04 - 0.04) | 395 (145-806) | 2.63 (0.97-5.38) | 820 (301-1672) | 2.67 (0.98-5.45) | 0.05 (0.04 - 0.06) |
| United States of America | 369235 (194240-649175) | 500.55 (263.32-880.05) | 399139 (212235-710754) | 487.99 (259.48-868.97) | -0.13 (-0.46 - 0.21) | 3156 (1142-6659) | 4.28 (1.55-9.03) | 3429 (1249-7288) | 4.19 (1.53-8.91) | -0.11 (-0.42 - 0.2) |
| United States Virgin Islands | 171 (104-272) | 409.93 (250.11-654.26) | 110 (66-177) | 415.63 (250.72-669.61) | 0.05 (0.04 - 0.06) | 1 (1-3) | 3.51 (1.29-7.15) | 1 (0-2) | 3.55 (1.29-7.24) | 0.05 (0.03 - 0.06) |
| Uruguay | 2989 (1828-4867) | 277.65 (169.79-452.07) | 2668 (1627-4347) | 280.37 (170.98-456.76) | 0.03 (0.03 - 0.04) | 24 (8-50) | 2.2 (0.74-4.62) | 21 (7-44) | 2.21 (0.74-4.64) | 0.03 (0.02 - 0.04) |
| Uzbekistan | 41436 (25859-66414) | 387.82 (242.03-621.59) | 52711 (32482-84641) | 400.91 (247.05-643.76) | 0.12 (0.1 - 0.13) | 325 (106-679) | 3.04 (1-6.35) | 413 (137-866) | 3.14 (1.04-6.59) | 0.12 (0.1 - 0.13) |
| Vanuatu | 175 (112-278) | 212.51 (136.51-337.5) | 301 (190-485) | 215.61 (136.46-348.18) | 0.05 (0.05 - 0.05) | 1 (0-3) | 1.68 (0.55-3.52) | 2 (1-5) | 1.7 (0.57-3.6) | 0.06 (0.05 - 0.07) |
| Venezuela (Bolivarian Republic of) | 36817 (22971-58482) | 404.92 (252.64-643.19) | 38541 (23309-61947) | 414.46 (250.66-666.16) | 0.08 (0.07 - 0.09) | 290 (96-603) | 3.19 (1.06-6.63) | 303 (101-637) | 3.26 (1.09-6.85) | 0.08 (0.07 - 0.08) |
| Viet Nam | 64529 (39014-106708) | 193.49 (116.98-319.96) | 55984 (33351-92186) | 199.42 (118.8-328.38) | 0.1 (0.09 - 0.12) | 508 (167-1070) | 1.52 (0.5-3.21) | 440 (144-935) | 1.57 (0.51-3.33) | 0.09 (0.08 - 0.11) |
| Yemen | 33182 (20990-53083) | 390.4 (246.95-624.53) | 66148 (41260-105582) | 405.45 (252.9-647.16) | 0.13 (0.12 - 0.14) | 260 (87-549) | 3.06 (1.02-6.46) | 520 (172-1091) | 3.19 (1.06-6.69) | 0.14 (0.12 - 0.15) |
| Zambia | 14642 (9251-23418) | 311.02 (196.52-497.45) | 30946 (19414-50183) | 316.16 (198.35-512.71) | 0.06 (0.05 - 0.06) | 125 (46-256) | 2.66 (0.98-5.44) | 264 (97-537) | 2.7 (0.99-5.48) | 0.05 (0.04 - 0.07) |
| Zimbabwe | 18760 (11606-30686) | 311.37 (192.63-509.32) | 24049 (14880-39233) | 315.56 (195.25-514.81) | 0.04 (0.03 - 0.05) | 160 (59-326) | 2.66 (0.99-5.41) | 206 (76-416) | 2.7 (1-5.46) | 0.06 (0.05 - 0.07) |

AAPC = average annual percent change; YLDs = years lived with disability.
